# Phase Transitions, Dielectric Response, and Nonlinear Optical Properties of Aziridinium Lead Halide Perovskites

**DOI:** 10.1021/acs.chemmater.3c02200

**Published:** 2023-11-14

**Authors:** Mirosław Mączka, Maciej Ptak, Anna Gągor, Jan K. Zaręba, Xia Liang, Sergejus Balčiu̅nas, Oleksandr A. Semenikhin, Olesia I. Kucheriv, Il’ya A. Gural’skiy, Sergiu Shova, Aron Walsh, Ju̅ras Banys, Mantas Šimėnas

**Affiliations:** † Institute of Low Temperature and Structure Research, Polish Academy of Sciences, ul. Okólna 2, 50-422 Wrocław, Poland; ‡ Institute of Advanced Materials, Faculty of Chemistry, Wrocław University of Science and Technology, 50-370 Wrocław, Poland; § Department of Materials, Imperial College London, South Kensington Campus, London SW7 2AZ, U.K.; ∥ Faculty of Physics, Vilnius University, LT-10257 Vilnius, Lithuania; ⊥ Department of Chemistry, Taras Shevchenko National University of Kyiv, 64 Volodymyrska St., Kyiv 01601, Ukraine; # Department of Inorganic Polymers, Petru Poni Institute of Macromolecular Chemistry, Aleea Grigore Ghica Voda 41-A, Iasi 700487, Romania; ∇ Department of Physics, Ewha Womans University, Seoul 03760, Korea

## Abstract

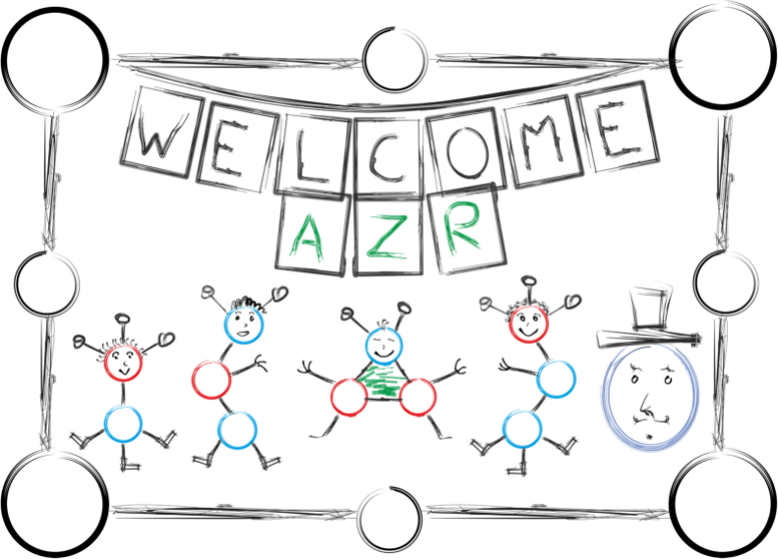

Hybrid organic–inorganic lead halide perovskites are promising candidates for next-generation solar cells, light-emitting diodes, photodetectors, and lasers. The structural, dynamic, and phase-transition properties play a key role in the performance of these materials. In this work, we use a multitechnique experimental (thermal, X-ray diffraction, Raman scattering, dielectric, nonlinear optical) and theoretical (machine-learning force field) approach to map the phase diagrams and obtain information on molecular dynamics and mechanism of the structural phase transitions in novel 3D AZRPbX_3_ perovskites (AZR = aziridinium; X = Cl, Br, I). Our work reveals that all perovskites undergo order–disorder phase transitions at low temperatures, which significantly affect the structural, dielectric, phonon, and nonlinear optical properties of these compounds. The desirable cubic phases of AZRPbX_3_ remain stable at lower temperatures (132, 145, and 162 K for I, Br, and Cl) compared to the methylammonium and formamidinium analogues. Similar to other 3D-connected hybrid perovskites, the dielectric response reveals a rather high dielectric permittivity, an important feature for defect tolerance. We further show that AZRPbBr_3_ and AZRPbI_3_ exhibit strong nonlinear optical absorption. The high two-photon brightness of AZRPbI_3_ emission stands out among lead perovskites emitting in the near-infrared region.

## Introduction

Hybrid lead halide perovskites have received enormous interest in recent years due to their multiple functional properties and rich structural diversity.^1−9^ One of the most important subgroups of lead halide perovskites constitutes three-dimensional (3D) analogues of the general formula APbX_3_ (A = Cs or organic cation, X = Cl, Br, I). These compounds can be obtained for only a handful of small organic cations, with the first example being the methylammonium (MA) analogue reported in 1978.^10^ Interest in these compounds increased dramatically after the discovery of their photovoltaic properties in 2009.^11^ Extensive searches for new 3D lead halide perovskites led to the discovery of formamidinium (FA) analogues in 2013.^12^ Later studies confirmed that both MAPbI_3_ and FAPbI_3_ are suitable materials for low-cost solar cell applications with power conversion efficiency currently exceeding 25%.^5,13^ The conventional MA- and FA-based perovskites are also prospective materials for many other applications, such as light-emitting diodes,^7^ photodetectors,^14,15^ and lasers.^15^ In 2020, the synthesis of 3D lead halide perovskites comprising methylhydrazinium (MHy^+^) was reported, opening up a new, unconventional subclass of 3D perovskites.^16,17^ In a similar manner to the MA and FA analogues, MHyPbX_3_ perovskites exhibited strong photoluminescence, which was also found effectively accessible through the two-photon excitation pathway.^16−19^ Nevertheless, one of the hallmark features of MHy^+^-based perovskites that sets them apart from conventional 3D perovskites is the general propensity to crystallize as polar structures below 400 K, even though MHy^+^ itself is not chiral.^16−18^ Indeed, both MHyPbBr_3_ and MHyPbCl_3_ feature second harmonic generation (SHG) activity at room temperature (RT), with the latter showing even a very rare SHG switching between two *on*-states at a high temperature (HT).

Based on theoretical calculations, Zheng and Rubel proposed in 2018 that cyclic AZR^+^ (CH_2_CH_2_NH_2_^+^) cations can be used for the construction of a stable AZRPbI_3_ perovskite, possibly suitable for photovoltaic applications.^20^ In 2022, theoretical predictions were confirmed by the synthesis of a stable 3D AZRPbX_3_ family, wherein X = Cl, Br, I.^21,22^ In particular, it has been shown that AZRPbX_3_ perovskites at RT structurally correspond to conventional MA and FA analogues (crystallize in the same *Pm*3̅*m* cubic setting).^21,22^ For unconventional MHy perovskites, the *Pm*3̅*m* cubic phase was reported for the HT phase of MHyPbBr_3_, while the chloride analogue maintains a polar *Pb*2_1_*m* structure up to the decomposition temperature.^16,17^ The optical studies revealed that band gaps of AZRPbX_3_ are narrower compared to the MA, FA, and MHy analogues, and that they feature very small exciton binding energies, attractive for photovoltaic applications.^22,23^ Preliminary optical studies have also shown intense narrow photoluminescence which may be relevant for light-emitting applications.^22,23^

It is well-known that the optoelectronic properties and stability of lead halide perovskites are affected by structural phase transitions (PTs), which alter the dynamics of the organic cations and distortion of the inorganic framework.^3,4,24−29^ In the case of MAPbX_3_ and FAPbX_3_ perovskites, the organic cations are disordered at RT, and on cooling they exhibit partial or complete ordering associated with a number of structural PTs, distortion of the inorganic framework, and decrease of crystal symmetry from cubic to tetragonal and then to the orthorhombic phase.^12,30−32^ An order–disorder PT was also reported for MHyPbBr_3_, whereas MHyPbCl_3_ showed a displacive-type transition from the *Pb*2_1_*m* to *P*2_1_ phase.^16,17,33^ A complete ordering of organic cations also leads to pronounced step-like dielectric anomalies, typical for switchable dielectric materials.^3,16,26,29,34,35^

Despite the significance of the PTs and the dielectric response in determining the performance of lead halide perovskites, these properties of the AZRPbX_3_ compounds have not yet been investigated. Herein, we report a multitechnique experimental and theoretical approach to identify the structural PTs in these compounds and elucidate their mechanisms. Our work reveals the presence of several transformations that significantly affect the structural, dielectric, and nonlinear optical (NLO) properties of these compounds.

## Experimental and Calculation Details

### Synthesis

AZRPbX_3_ powders and single crystals were obtained as described previously.^21^ The toxicity of the volatile aziridine should be properly taken into account in the synthesis of the title perovskites.

### Powder X-ray Diffraction

Powder X-ray diffraction (PXRD) experiments were carried out using the Bragg–Brentano geometry and 2Theta-Omega scans on a Panalytical powder diffractometer. To maintain a low temperature during the measurements, we utilized an Oxford PheniX cryostat. The radiation source employed was the Cu Kα1,2 doublet. For the Rietveld refinement of the collected data, the Jana2006 program was employed.^36^ The crystallographic data of the structures from PXRD were deposited with the Cambridge Crystallographic Data Center (CCDC 2285006 for AZRPbBr_3_ and 2285007 for AZRPbI_3_).

### Single-Crystal X-ray Diffraction

Single-crystal X-ray diffraction (SCXRD) data were collected at 100 K using an Oxford-Diffraction XCALIBUR Eos CCD diffractometer with graphite-monochromated Mo-Kα radiation. The unit-cell determination and data integration were carried out using the CrysAlisPro package from Oxford Diffraction. Room-temperature crystal structures of all three perovskites were cubic and did not display any signs of twinning. On the other hand, upon transition to low-temperature phases, there is always a transition to multicomponent twins of lower symmetry. Only in the case of the bromide perovskite were we able to identify the symmetry of the low-temperature phase from a single-crystal experiment, since this indexing process is highly disturbed by the twinning. Twin components in the AZRPbBr_3_ were identified using CrysAlisPro software (four components cover 99% reflexions) and the hklf5-format file was created upon reduction. Twin lattices along major crystallographic directions are shown in Figures S1–S3. The structures were solved with ShelXT program using an intrinsic phasing method and refined by a full-matrix least-squares method on F^2^ with ShelXL.^37,38^ Olex2 was used as an interface to the ShelX programs.^37^ Pb, Br, and C|N atoms were refined anisotropically. C and N atoms were set to have the same coordinates and atomic displacement parameters (ADPs). H atoms were positioned geometrically and were not refined. Analytical numeric absorption correction using a multifaceted crystal model was applied.^39^ The crystallographic data of the AZRPbBr_3_ structure from SCXRD data were deposited with the Cambridge Crystallographic Data Center (CCDC 2270772).

### DSC

Differential scanning calorimetry (DSC) measurements were performed using Linkam DSC 600 stage operating at a scan rate of 10 K min^–1^ in the temperature range of 90–280 K. The samples were placed in a chamber at RT, which was purged with dry nitrogen for 10 min prior to each measurement. Masses of the used samples were 51.2, 48.6, and 70.7 mg for AZRPbCl_3_, AZRPbBr_3_, and AZRPbI_3_, respectively. The obtained data were processed using LINK and OriginPro software.

### Raman Studies

Temperature-dependent Raman spectra of AZRPbBr_3_ and AZRPbCl_3_ samples in the 1600–100 cm^–1^ range were measured using a Renishaw inVia Raman spectrometer equipped with a confocal DM2500 Leica optical microscope and a thermoelectrically cooled CCD detector. Excitation was performed by using a diode laser operating at 830 nm, and the spectral resolution was 2 cm^–1^. The low-wavenumber range (300–10 cm^–1^) was measured on the same spectrometer using an Eclipse filter. The temperature was controlled using Linkam THMS600 stage.

### Dielectric Studies

Dielectric spectroscopy experiments of pressed pellet samples were performed in the 20 Hz–1 MHz frequency range using an HP4284A LCR meter. The flat capacitor model was used to calculate the complex dielectric permittivity from the measured capacitance and dielectric loss tangent. Silver paste was used as a sample electrode. We did not observe any indications of silver halide formation as the color of the electrode did not change and was typical for the silver paste. Furthermore, we also did not observe any unexpected behavior in the dielectric response, which could signify the formation of such a phase. Temperature-dependent dielectric spectra were measured on cooling at a rate of 1 K/min.

### Nonlinear Optical Studies

Nonlinear optical experiments were performed using a laser system employing a wavelength-tunable Topaz Prime Vis–NIR optical parametric amplifier (OPA) pumped by a Coherent Astrella Ti:sapphire regenerative amplifier providing femtosecond laser pulses (800 nm, 75 fs) at a 1 kHz repetition rate. Experiments employing 1300 nm laser pulses used the attenuated output of a tunable OPA. Experiments employing 800 nm laser pulses used the output of a regenerative amplifier passed through a 5 mm aperture. Laser fluence at samples was equal to 0.21 mJ/cm^2^ (1300 nm) and 0.30 mJ/cm^2^ (800 nm). The single crystals of AZRPbCl_3_, AZRPbBr_3_, and AZRPbI_3_ were crushed with a spatula and sieved through an Aldrich minisieve set, collecting a microcrystal size fraction of 63–88 μm. The size-graded samples were fixed in-between microscope glass slides to form tightly packed layers, sealed, and mounted to the horizontally aligned sample holder. No refractive index matching oil was used. The employed measurement setup operates in the reflection mode. Specifically, the laser beam was directed onto the sample at 45° to its surface. Emission collecting optics consisted of a Ø25.0 mm plano-convex lens of a 25.4 mm focal length mounted to a 400 μm 0.22 NA glass optical fiber, which was placed along the normal to the sample surface. The distance between the collection lens and the sample was equal to 30 mm. The spectra of the temperature-dependent NLO responses were recorded by an Ocean Optics Flame T XR fiber-coupled CCD spectrograph with a 200 μm entrance slit. Scattered pumping radiation was suppressed with the use of a Thorlabs 750 nm short-pass dielectric filter (FESH0750) for studies on AZRPbCl_3_ and AZRPbBr_3_, and a 1100 nm short-pass dielectric filter (FESH1100) in the case of AZRPbI_3_. Temperature control of the sample was performed using a Linkam LTS420 Heating/Freezing Stage and the measurements were performed in a temperature range of 93–293 K upon heating and cooling runs with a constant d*T*/d*t* of 10 K min^–1^. Temperature stability was equal to 0.1 K. The same optical setup was employed for power-dependent studies and determination of the two-photon brightness of AZRPbBr_3_ and AZRPbI_3_ samples.

The two-photon brightness values for AZRPbBr_3_ (800 nm) and AZRPbI_3_ (1300 nm) were determined using the solid-state two-photon excited fluorescence technique (SSTPEF). Prior to SSTPEF measurements, the solid samples of AZRPbBr_3_, AZRPbI_3_, and reference compounds (bis(4-diphenylamino)stilbene (BDPAS) and Styryl 9M) were finely crushed and fixed between microscope glass slides. The two-photon upconverted emissions were excited using the same beam and geometrical parameters. Densities of AZRPbBr_3_ and AZRPbI_3_, necessary for the calculation of two-photon brightness values, were taken from the room-temperature crystallographic data.

### Molecular Dynamics

Molecular dynamics (MD) trajectories of AZRPbX_3_ (X = I, Br, Cl) were simulated using a machine-learning force field (MLFF).^40^ The MLFFs were trained using an on-the-fly approach based on the Gaussian process regression with Bayesian error estimation as implemented within the Vienna Ab initio Simulation Package (VASP).^41,42^ Force fields were trained individually for each material, and the training data sets were generated using 104-atom supercell NpT MD simulations with an external pressure of 1 bar, where on-the-fly local configuration selection was performed. A Langevin thermostat was applied, and the atomic and lattice friction constants were both 10 ps^–1^. The MD time step was set to 0.5 fs. The energy, forces, and stress tensor of each selected frame were computed based on density functional theory (DFT), where the r^2^SCAN exchange-correlation functional was used. The plane wave basis set cutoff energy was 500 eV. For all three compositions, the configuration collection was performed on 100, 200, and 300 K with constant-temperature MD. Then, the trajectories for property analysis were produced based on the trained force fields on a 2808-atom supercell with the same MD settings as the training step except that the time step was increased to 1 fs. The production of MD trajectories was performed again with constant-temperature NpT MD at temperatures between 80 and 300 K to investigate the equilibrated dynamic properties in this temperature range.

The output MD trajectories were analyzed with the Perovskite Dynamics Analysis (PDynA) package,^43^ which is an integrated Python code for analyzing atomistic MD trajectories of perovskite materials that can extract dynamic information and correlations of octahedra and A-site molecules, and can output octahedral tilting and distortion, local lattice parameters, A-site molecular orientations, etc. This method has been previously tested on CsPbI_3_ and MAPbBr_3_ and further details of force field training, molecular dynamics, and trajectory analysis can be found in ref (43)(43).

### Molecular Rotations

The molecular cation rotation energy barrier was calculated with single-point DFT energy evaluations, where the molecules were rotated with respect to their center of mass about the three principal axes in a 104-atom supercell. The total energy (per formula unit) was computed for 0–360° rotation with an interval of 5°. The PBEsol^44^ exchange-correlation functional was selected, and projector augmented wave potentials were used with all projection operators evaluated in reciprocal space. The plane wave basis set cutoff energy was set to 600 eV. A Gaussian smearing with a width of 50 meV was adopted for the smearing of the electronic band occupancy. A 2 × 2 × 2 Γ-centered *k*-point grid was used for all calculations.

## Results and Discussion

### DSC

**Figure 1:**
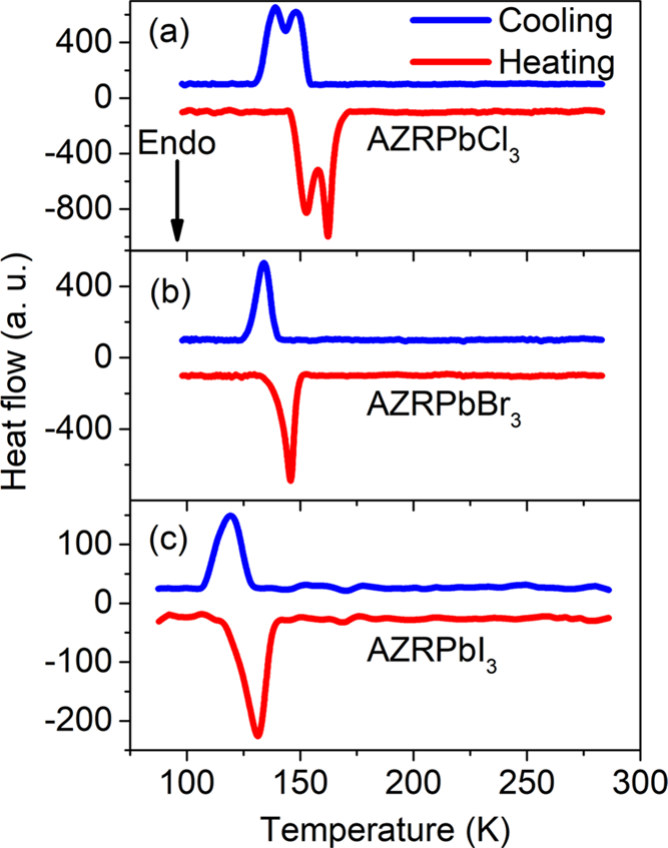
DSC curves of (a) AZRPbCl_3_, (b) AZRPbBr_3_, and (c) AZRPbI_3_ measured during the heating and cooling runs.

First, we determined the PT points in the AZRPbX_3_ compounds using the DSC experiments. The DSC measurements of AZRPbCl_3_ show the presence of two closely spaced heat anomalies at *T*_1_ = 162 K (149 K) and *T*_2_ = 154 K (139 K) observed during heating (cooling) (Figure 1a and Table 1). Large thermal hysteresis is consistent with the first-order character of both PTs. The associated changes in enthalpy Δ*H* and entropy Δ*S* are ∼1.12 kJ mol^–1^ and ∼7.2 J mol^–1^ K^–1^ for the PT at *T*_1_, respectively, and ∼1.4 kJ mol^–1^ and ∼9.65 J mol^–1^ K^–1^ for the PT at *T*_2_ (average values). Based on the Δ*S* = *R* ln(*N*) equation for an order–disorder phase transition, where *R* is the gas constant, and *N* is the ratio of the number of configurations in the disordered and ordered phases, the *N*_1_ and *N*_2_ values were calculated as 2.38 and 3.19. The large values of *N*_1_ and *N*_2_ are consistent with the order–disorder character of both PTs. Note that the PT temperatures and entropies are slightly lower compared to the *Pm*3̅*m* → *P*4/*mmm* → *P*222_1_ PT sequence of the related MAPbCl_3_ perovskite (*T*_1_ = 177.2 K and Δ*S* = 10.0 J mol^–1^ K^–1^; *T*_2_ = 171.5 K and Δ*S* = 14.6 J mol^–1^ K^–1^).^45,46^ The PT temperatures are also much lower than for the FAPbCl_3_, where the *Pm*3̅*m* → *P*4/*mbm* → *Cmcm* symmetry change is observed at 271 and 258 K.^47^

**Table 1:** Temperatures of PTs and Thermodynamic Retrieved Parameters from DSC Measurements

	heating mode				cooling mode			
	*T*_PT_ (K)	Δ*H* (kJ mol^–1^)	Δ*S* (J mol^–1^ K^–1^)	*N*	*T*_PT_ (K)	Δ*H* (kJ mol^–1^)	Δ*S* (J mol^–1^ K^–1^)	*N*
AZRPbCl_3_	154	1.46	9.5	3.14	139	–1.34	–9.8	3.25
	162	1.2	7.4	2.44	149	–1.04	–7.0	2.32
AZRPbBr_3_	145	1.24	8.5	2.78	134	–1.21	–9.0	2.95
AZRPbI_3_	132	0.54	4.1	1.64	119	–0.53	–4.5	1.72

DSC measurements of AZRPbBr_3_ show the presence of only one heat anomaly at *T*_1_ = 145 K (134 K) with a significant hysteresis suggesting the first-order character of the PT (Figure 1b). The corresponding changes in enthalpy Δ*H* and entropy Δ*S* are ∼1.23 kJ mol^–1^ and ∼8.8 J mol^–1^ K^–1^ (average values), and the calculated value of *N* is 2.86. This behavior significantly differs from that observed for the related MAPbBr_3_, which exhibits three PTs, associated with *Pm*3̅*m* → *I*4/*mcm* → *P*4/*mmm* → *Pnma* symmetry lowering,^4,27,46,48^ occurring at *T*_1_ = 236.3 K (Δ*S* = 8.2 J mol^–1^ K^–1^), *T*_2_ = 154.0 K (Δ*S* = 4.1 J mol^–1^ K^–1^), and *T*_3_ = 148.8 K (Δ*S* = 11.2 J mol^–1^ K^–1^).^45^ A very different sequence of PTs was also reported for FAPbBr_3_, i.e., *Pm*3̅*m* → *P*4/*mbm* → *P*4/*mbm* → *P*4/*mbm* → *Pnma* → *Pnma* at 266, 182, 162, 153, and 118 K, respectively.^32^

DSC measurements of AZRPbI_3_ reveal only one heat anomaly at *T*_1_ = 132 (119 K) (Figure 1c). The associated changes in thermodynamic parameters are Δ*H* ∼ 0.54 kJ mol^–1^ and Δ*S* ∼ 4.3 J mol^–1^ K^–1^ (average values), while *N* is 1.68. The behavior of AZRPbI_3_ is also different from the related FAPbI_3_, which undergoes much more complicated *Pm*3̅*m* → *I*4/*mbm* → *I*4/*mbm* symmetry lowering at 285 and 140 K,^49^ and MAPbI_3_, for which two PTs associated with the *Pm*3̅*m* → *I*4/*mcm* → *Pnma* symmetry change^31^ lead to much larger entropy changes (Δ*S* = 9.7 J mol^–1^ K^–1^ at *T*_1_ = 330.4 K and Δ*S* = 19.0 J mol^–1^ K^–1^ at *T*_2_ = 161.4 K).^45^

In general, the DSC results of the AZRPbX_3_ compounds show two trends. First, the value of *N* exhibits a large decrease in the order 5.57 (Cl) > 2.86 (Br) > 1.68 (I), suggesting a decreasing contribution of the ordering/disordering processes to the mechanism of the PTs. Second, the temperatures of the *T*_1_ phase transitions decrease in the same order (Cl > Br > I), suggesting the highest cubic phase stability for the iodide compound. The cubic phases of AZRPbX_3_ are also stabilized at much lower temperatures compared to the MAPbX_3_ and FAPbX_3_ analogues, which is especially pronounced for bromides and iodides.

### X-ray Diffraction

At RT, all three perovskites crystallize in the cubic *Pm*3̅*m* space group as was found previously in SCXRD experiments.^21^ Their structural motive comprises regular PbX_6_ octahedra connected in a corner-sharing manner; namely, all X–Pb–X angles are 90° and all Pb–X bonds are equal. AZR^+^ cation is highly disordered in the cubic phase. Upon transitions to the low-temperature (LT) phases, there are two major structural perturbations possible: (i) deformation of the PbX_6_ framework geometry and (ii) ordering of the AZR^+^ cations. Structural analysis using single crystals and powder samples was further performed to analyze these structural changes.

**Figure 2:**
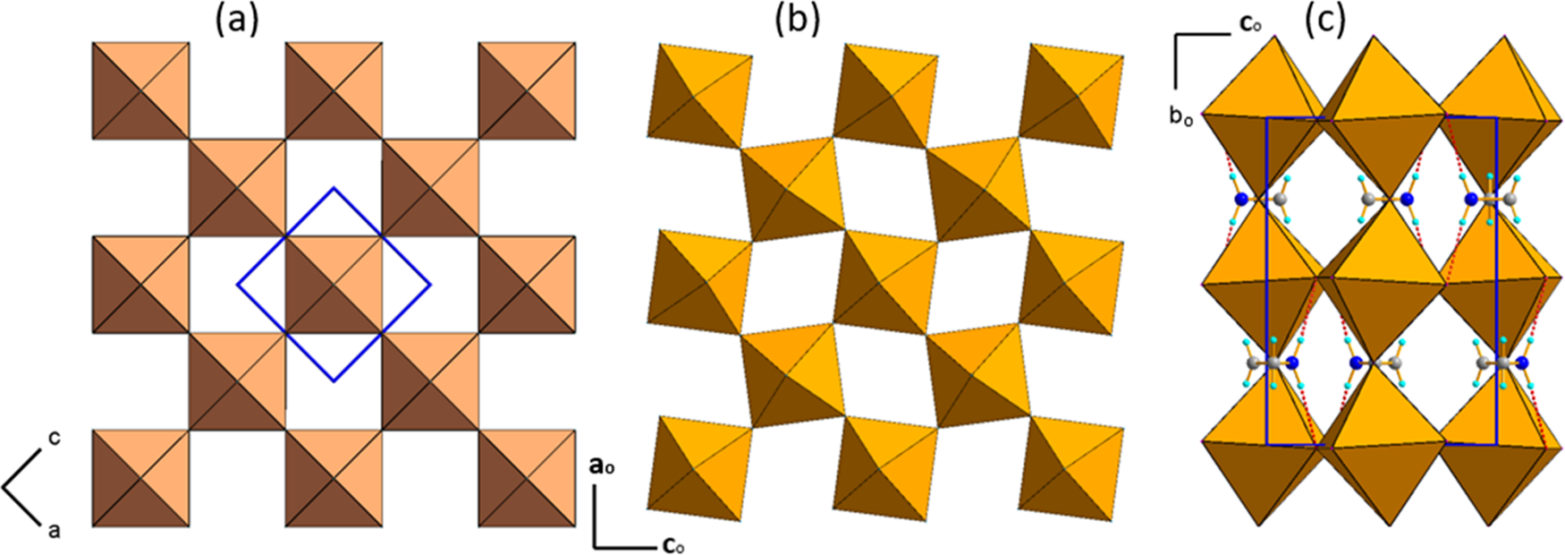
Structure of (a) cubic aristo-phase with the marked unit cell and (b) LT phase of the inorganic framework of AZRPbI_3_ with *a*^*–*^*b*^*+*^*a*^*–*^ tilting, (c) unit-cell packing in the *Pnma* phase of AZRPbI_3_ with AZR^+^ placed in the (*a*_*o*_*,c*_*o*_) planes, dashed red lines stand for HBs.

By examining the powder diffraction patterns of AZRPbI_3_, we observed that this compound transforms at low temperature to orthorhombic *Pnma* symmetry with the unit-cell dimensions *a* = 8.878(1) Å, *b* = 12.657(1) Å, and *c* = 8.859(1) Å similar to the orthorhombic polymorph of MAPbI_3_ matching the (1,0,1), (0,2,0), and (−1,0,1) lattice transformation with respect to the cubic *Pm*3̅*m* aristotype.^50,51^ The Rietveld refinement results for powders measured at 20 K are shown in Figure S4. The diffractograms of the HT cubic *Pm*3̅*m* parent phase and the LT orthorhombic modification are shown in Figure S5. The crystal structure of AZRPbI_3_ is presented in Figure 2a–c. The out-of-phase rotations of the octahedra correspond to the *a*^–^*b*^+^*a*^–^ tilt system similar to the LT phases of MAPbI_3_ and CsPbI_3_.^52,53^ One of the possible placements of AZR^+^ cations which gives the ordered molecular substructure is shown in Figure 2c. The distribution of AZR^+^ in the (*a,c*) planes allows for the formation of N–H···I hydrogen bonds with a N···I distance of 3.67 Ȧ and N–H–I angle of 165°.

**Figure 3:**
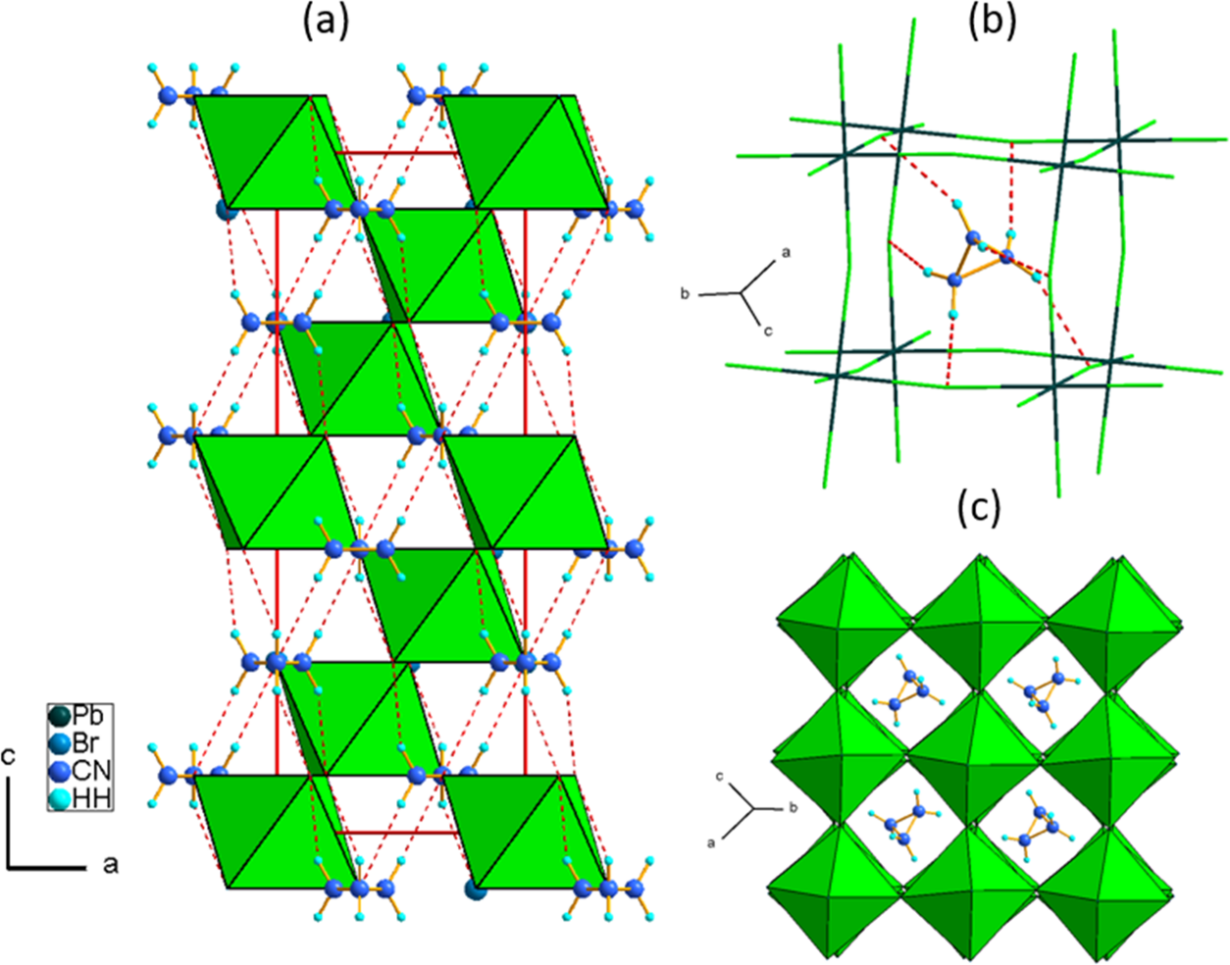
LT (12 K) crystal structure of AZRPbBr_3_ obtained using the PXRD experiment: (a) Crystal packing along with possible N–H···Br HBs (red dashed lines); (b) AZR^+^ placement in the perovskite cavity; and (c) general view along the [100] cubic direction.

The LT crystal structure of AZRPbBr_3_ was investigated at 100 K using the SCXRD experiments and at 12 K using PXRD. Both experiments confirm the trigonal system of the *R*3̅*c* space group with *a* ∼ 8.49 Å, *c* ∼ 20.07 Å, and γ = 120° (at 100 K). The crystallographic data from SCXRD are summarized in Tables S1–S3, whereas the results of Rietveld refinement are presented in Figure S6. The temperature changes of the diffraction patterns due to symmetry reduction from cubic *Pm*3̅*m* to the LT polymorph are shown in Figure S7. In the model derived from SCXRD, Br^–^ anions were refined as disordered between two positions (Pb–Br(1) = 3.013(12) Å, Pb–Br(1)^i^ = 2.969(12) Å), symmetry code (i) 2/3 – *y* + *x*, 4/3 – *y*, 5/6 – *z*) with the 0.5 occupancy to eliminate the large ADP max/min of ca. 4.1 prolate. On the other hand, refinement of powder diffraction data gives satisfactory results for all ordered bromine ions. Possibly, the disorder observed in SCXRD arises from the complex twinning of the sample and bias from the contribution of four domain states to the diffracted intensities especially because refinements in lower symmetry (*R*3*c* and *I*2/*a*) also showed the disorder around Br(1) position. The packing of the LT phase of AZRPbBr_3_ is shown in Figure 3a.

The transition of the PT to the LT phase is also followed by the ordering of AZR^+^ cations, which are located in the voids of the 3D framework (Figure 3b). Cations lay on the 3-fold axis, C and N atoms of the cation are not distinguished in this model and occupy the same crystallographic position (C–C|N = 1.53(5) Å). The organic cations create a set of H-bonds with the inorganic framework with the shortest N···Br distance of 3.50(2) Å at 100 K (Figure 3b). The symmetry reduction from cubic *Pm*3̅*m* to trigonal *R*3̅*c* leads to *a*^*–*^*a*^*–*^*a*^*–*^ tilting (Figure 3c).

Determining the crystal structure of the LT phase of AZRPbCl_3_ proved to be challenging. Despite our efforts, we were unable to derive a structural model from the heavily twinned single crystal or index the powder diagram based on the known orthorhombic LT structures reported for MAPbCl_3_^54,55^ and FAPbCl_3_.^35^ Interestingly, we were able to index the entire diffraction patterns using a monoclinic unit cell, similar to those found in heavily distorted MHyPbCl_3_ (with dimensions *a* = 11.74 Ȧ, *b* = 10.75 Å, *c* = 5.71 Å, β = 92.7°).^17^ However, our attempts to refine the model were unsuccessful. This suggests that AZRPbCl_3_ adopts a distinct LT structure which is further supported by the compatibility of the diffraction pattern with the monoclinic distortion of the γ-phase of CsPbCl_3_, featuring dimensions *a* = 11.06 Å, *b* = 7.56 Å, *c* = 8.66 Å, and β = 92.7°.^56,57^ It is worth noting that the monoclinic *P*2_1_/*m* phase was postulated for CsPbCl_3_ based on the neutron diffraction studies.^58^Figure S8 illustrates the X-ray powder diffraction patterns of AZRPbCl_3_ at 200 and 100 K, evidencing a substantial symmetry breaking at a low temperature.

### Materials modeling

**Figure 4:**
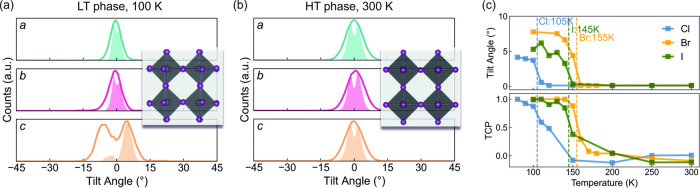
Octahedral tilting in (a) LT phase at 100 K and (b) HT cubic phase at 300 K of AZRPbI_3_. Each panel corresponds to one axis. The solid lines denote the dynamic distribution of tilting. The shaded area below the solid lines is the correlation of the tilt angle with the next nearest neighbor along the same direction. The corresponding global Glazer tilting pattern is *a*^0^*a*^0^*c*^*+*^ and *a*^0^*a*^0^*a*^0^. (c) *z*-tilt angle (upper panel) and tilt correlation polarity (TCP, lower panel) of AZRPbX_3_ versus temperature.

We used MD simulations, based on MLFF forces, to obtain additional details on the structural PTs and dynamics of AZRPbX_3_ compounds. For all three halides, our simulations provided two structural phases (HT cubic and LT tetragonal-like) evident by the distinct octahedral tilting patterns extracted from the equilibrated molecular dynamics trajectories with PDynA.^43^Figure 4 illustrates the distribution of the dynamic tilting of octahedra in AZRPbI_3_. At 100 K (Figure 4a), an approximately 5° tilt is found in one of the principal axes; along this axis, all octahedra tilt in the same direction (Glazer notation of *a*^0^*a*^0^*c*^*+*^). In contrast, at 300 K (Figure 4b), the tilting in all three directions is zero on average, leading to a cubic phase with a Glazer notation of *a*^0^*a*^0^*a*^0^. Similar tilting modes are simulated for AZRPbBr_3_ and AZRPbCl_3_, with slightly different tilt angles and PT temperatures as shown in Figure 4c.

We define the structural PT temperature as the temperature at which a significant nonzero tilt angle occurs in the *z*-axis when the temperature is lowered. The simulated PT temperatures of AZRPbI_3_, AZRPbBr_3_, and AZRPbCl_3_ are 145, 155, and 105 K, respectively (Figure 4c), which are reasonably close to the experimental values. It is noteworthy that the tilting correlation polarity (TCP) increases from 0 (no preferred correlation, characteristic value of cubic phase) toward 1 (perfect in-phase correlation, characteristic value of tetragonal phase). This implies that even when the material is in the (average) cubic phase, when the temperature is approaching the PT temperature, the octahedra will have a preferred in-phase alignment along one axis (here in the *z*-direction). This local symmetry breaking effect of cubic phase under cooling is also found in MAPbBr_3_ but is not present in inorganic halide perovskites such as CsPbI_3_.^43,59^

**Figure 5:**
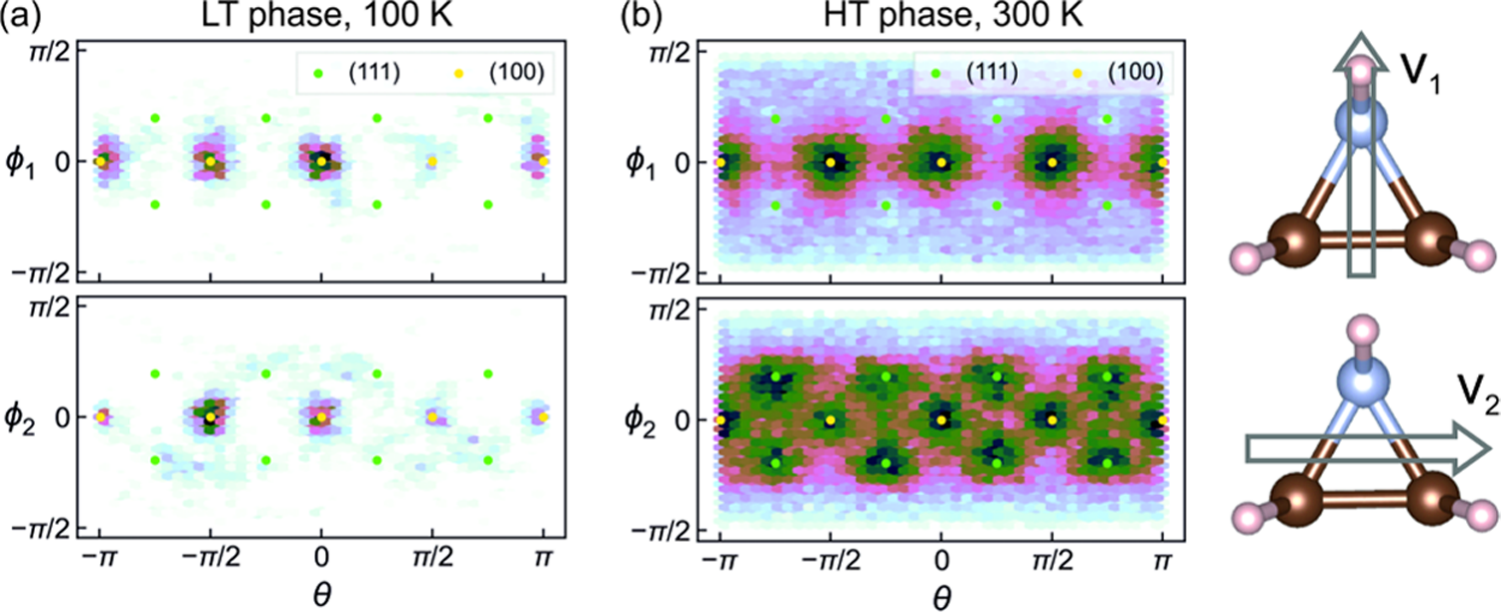
AZR^+^ orientation distribution in spherical coordinates in (a) LT phase at 100 K and (b) HT cubic phase at 300 K of AZRPbI_3_. The orientations of the AZR^+^ cation are projected onto the horizontal (azimuthal angle) and vertical (polar angle) axes. The upper panel in each subplot denotes the direction of the polarization vector v_1_, while the lower panel denotes the secondary vector v_2_, as visualized on the right.

We also simulated the preferred orientations of the AZR^+^ cations in the A-site. Two vectors are required to fully describe the orientation of an AZR^+^ in 3D, the first of which is the polarization vector v_1_, and the second vector v_2_ connects the two carbon atoms, as shown in Figure 5. In the tetragonal phase at 100 K, both v_1_ and v_2_ mostly point along the [100] family of directions (as shown in Figure 5a). This means that the plane formed by the carbon and nitrogen atoms in the AZR^+^ cation is very likely to be parallel to the *xy*-plane. It is noteworthy that this is different from the SCXRD experiment of AZRPbBr_3_, as the aziridinium molecular orientation shown in Figure 3 is associated with the *a*^*–*^*a*^*–*^*a*^*–*^ tilting mode, while Figure 5a illustrates the counterpart for *a*^0^*a*^0^*c*^*+*^ AZRPbI_3_. In the cubic phase at 300 K (Figure 5b), v_1_ has a preferred orientation along the [100] or equivalent direction, and v_2_ points along mostly the [111] and partially the [100] directions. The different symmetry of each phase is clearly reflected in the pattern of the preferred molecular orientations.

We also used single-point DFT to calculate the potential energy surface for the AZR^+^ orientation in the structure. The 4-fold symmetry in the energy landscape of molecular rotation is found for all compounds along all three lattice directions, as shown in Figure S9 for AZRPbBr_3_. The obtained energy barriers are summarized in Table 2, revealing an increase in the order Cl > Br > I. This increase is in agreement with the increase of the PT temperatures in the same order revealed by the DSC studies.

**Table 2:** Rotational Energy Barrier for Each Rotation Mode of AZRPbX_3_ Compounds Obtained from DFT Calculations

composition	rotation barrier (meV)		
	*a*-axis	*b*-axis	*c*-axis
X = I	89	113	170
X = Br	93	128	167
X = Cl	82	165	191

### Raman Studies

**Figure 6:**
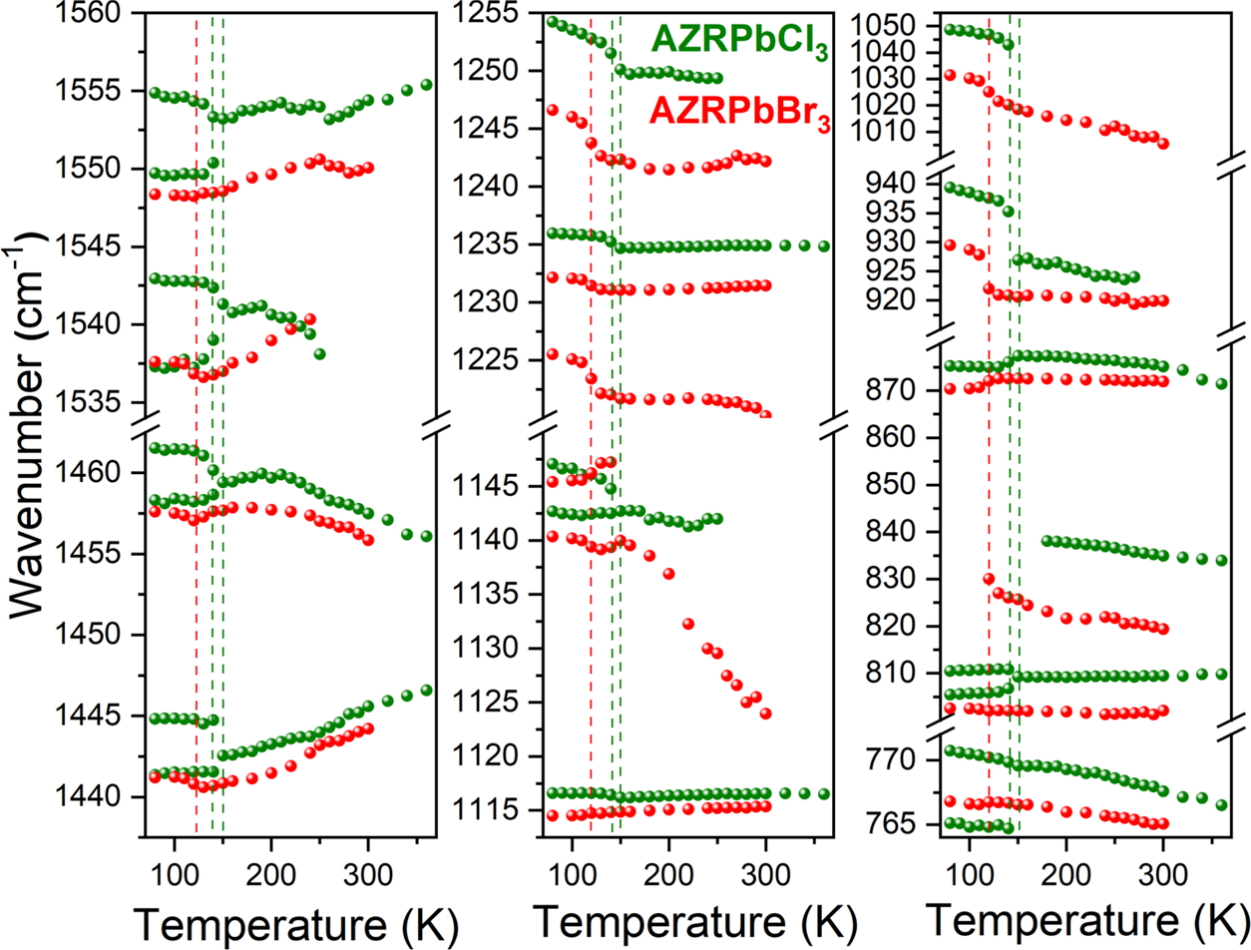
Temperature dependence of the Raman mode wavenumbers for AZRPbBr_3_ (red symbols) and AZRPbCl_3_ (green symbols). Vertical lines denote the PT temperatures.

**Figure 7:**
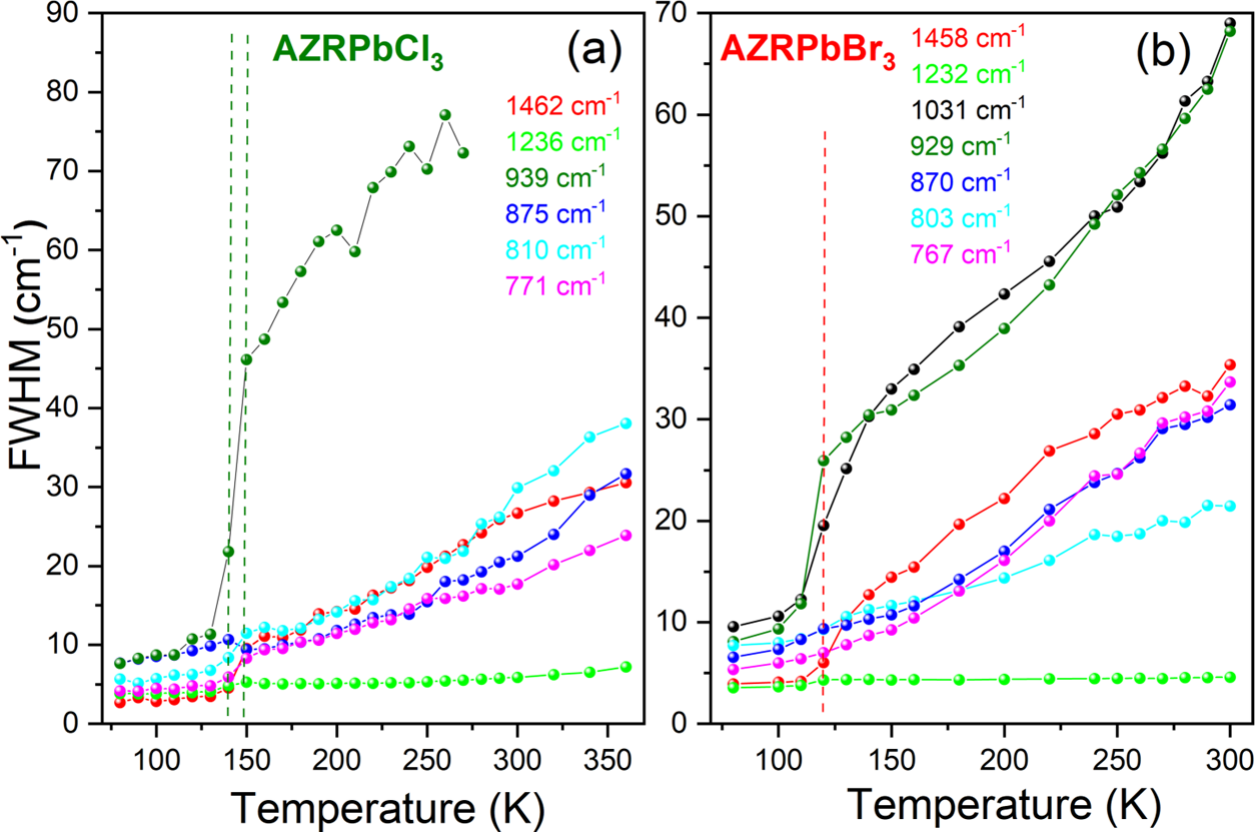
Temperature dependence of the Raman mode FWHM of (a) AZRPbCl_3_ and (b) AZRPbBr_3_. Vertical lines denote PT temperatures.

In order to obtain further insight into the PT mechanism, molecular cation dynamics, and phonon properties, we performed temperature-dependent Raman experiments for AZRPbCl_3_ and AZRPbBr_3_ (Figures S10–S13). Similar experiments for AZRPbI_3_ failed due to a very strong photoluminescence background. Plots of wavenumbers and full width at half-maximum (FWHM) values vs temperature are presented in Figures 6 and 7, respectively. The observed modes are listed in Table S4 together with the assignment based on previous RT Raman scattering studies of AZRPbX_3_ and temperature-dependent studies of MAPbX_3_, FAPbX_3_, and MHyPbX_3_ perovskites.^22,27,28,33,60,61^

Upon a temperature decrease, the majority of Raman bands related to the AZR^+^ vibrations shift to higher wavenumbers for both compounds (Figure 6). Furthermore, they exhibit narrowing, especially pronounced for the NH_2_-related bands observed near 1048–1005 cm^–1^ (ω(NH_2_)) and 939–920 cm^–1^ (ρ(NH_2_)) (Figures 7, S10, and S12). This behavior is consistent with the slowing of the reorientational motion of AZR^+^ cations in the *Pm*3̅*m* phase.

When the temperature decreases below 130 K, almost all internal modes of AZRPbBr_3_ exhibit clear shifts to higher wavenumbers (Figure 6) due to the onset of the structural PT. The lack of splitting of these modes is consistent with the SCXRD data, which revealed the presence of only one crystallographically unique AZR^+^ cation in the LT *R*3̅*c* phase. The wavenumber shift is accompanied by a large step-like decrease of the FWHM, especially pronounced for the bands corresponding to the ω(NH_2_) and ρ(NH_2_) modes (Figure 7b). This behavior proves that the PT is associated with the ordering of AZR^+^ cations, which is in agreement with the DSC and X-ray diffraction data. Note however that according to the X-ray diffraction data, the ordering of AZR^+^ cations is not complete, since, although these cations occupy the same crystallographic position, they present a 3-fold disorder in the LT *R*3̅*c* phase.

Pronounced narrowing of bands is also observed for AZRPbCl_3_ (Figures 6, 7, and S12). However, internal modes of this compound exhibit clear splitting into doublets with magnitudes up to 7 cm^–1^. Raman data indicate, therefore, the presence of two unique AZR^+^ cations in the LT phase of AZRPbCl_3_. It is worth noting that this type of behavior was not reported for MAPbX_3_ or FAPbX_3_ perovskites, but two distinct A-site cations in the LT phase were reported for MHyPbX_3_ polar analogues and the monoclinic γ-phase or *P*2_1_/*m* phase of CsPbCl_3_.^22,26−28,33,56−61^ Thus, Raman spectra support the conclusion derived from the powder X-ray diffraction data that the LT structure of AZRPbCl_3_ may be similar to the monoclinic structure of CsPbCl_3_.

The Raman spectra show that the only bands that do not show pronounced narrowing on cooling are those near 470 and 310 cm^–1^ for AZRPbCl_3_ and AZRPbBr_3_, respectively (Figures S10 and S12). This behavior confirms our previous assignment of these bands to the AZR-cage modes, since a lack of significant narrowing was previously reported also for the MA-, FA-, and MHy-cage modes.^27,28,60^ The remaining Raman bands observed below 200 cm^–1^ provide information about the structural changes of the inorganic framework. Figures S11 and S13 show that these modes exhibit pronounced narrowing and splitting below the PT temperatures, in agreement with the ordered AZR^+^ cations and decrease of crystal symmetry due to pronounced change in the distortion and tilts of the PbX_6_ octahedra. A larger number of bands observed at 80 K for AZRPbCl_3_ compared to AZRPbBr_3_ suggest lower symmetry and/or stronger distortion of the chloride framework. This behavior is consistent with the monoclinic symmetry postulated for AZRPbCl_3_.

### Dielectric Studies

**Figure 8:**
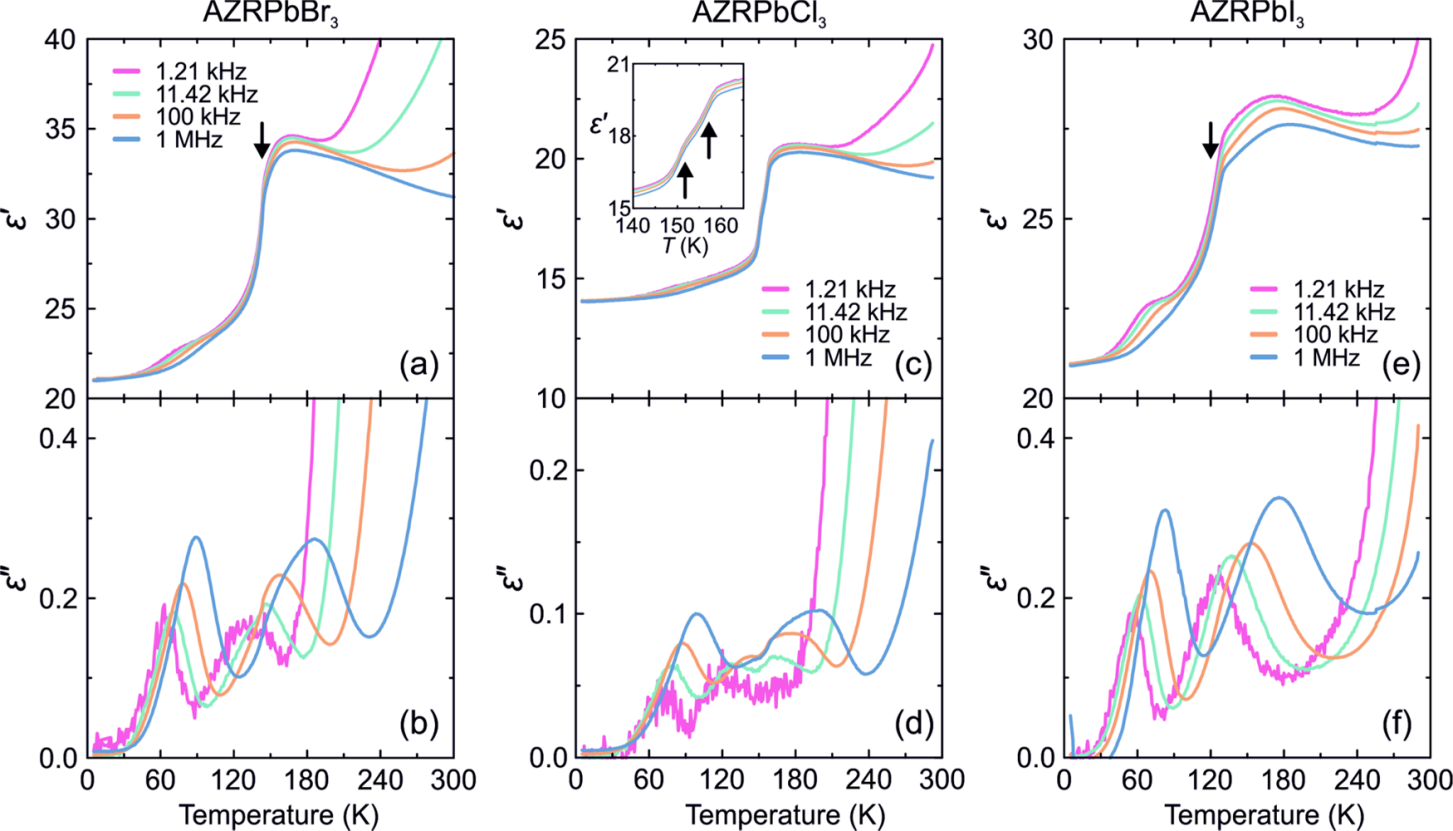
Temperature dependence of the real and imaginary parts of the complex dielectric permittivity of (a, b) AZRPbBr_3_, (c, d) AZRPbCl_3_, and (e, f) AZRPbI_3_ pellet samples obtained on cooling. Inset in panel (c) shows the anomaly associated with the two PTs occurring at about 156 and 151 K. Arrows indicate PT anomalies.

To further investigate the dynamics of AZR^+^ cations, we performed broad-band dielectric spectroscopy experiments on powdered samples. The temperature dependence of the real ε′ and imaginary ε″ parts of the complex dielectric permittivity (ε* = ε′ – iε″) of the AZRPbBr_3_ pellet sample is presented in Figure 8a,b. An anomalous step-like decrease of ε′ can be observed at about 145 K (Figure 8a) corresponding to the PT point, which is in good agreement with the DSC and Raman results. In addition to the PT anomaly, the complex dielectric permittivity of AZRPbBr_3_ shows at least three dielectric dispersions, which are best visible as peaks in the ε″ data (Figure 8b). Note that a very similar temperature evolution of ε* was also observed for the related MAPbX_3_ compounds.^3,4,26,29^

In addition to the PT anomaly, the complex dielectric permittivity of AZRPbBr_3_ shows at least three dielectric dispersions, which are best visible in the ε″ data (Figure 8b). The dispersion close to the RT originates from conductivity processes and is typical for hybrid lead halide perovskites.^62^ Two additional dielectric relaxations due to the dynamics of electric dipoles can be observed in the lower temperature region, with one of them crossing the PT anomaly and quickly disappearing (Figure 8b).

We characterized the relaxation times τ and activation energies *E*_a_ of these dipolar processes by analyzing the frequency domain data of ε″ (see Supporting Information (SI) for more details; Figures S14 and S15). The activation energy of the dipolar relaxation occurring above the PT point is 157(5) meV, which roughly doubles (*E*_a_ = 296(5) meV) as the PT point is crossed. This indicates a strong effect of the PT on the dipolar dynamics in AZRPbBr_3_. The activation energy of the dielectric relaxation occurring solely in the LT phase is 101(5) meV. Based on the similarities with the related MAPbX_3_ perovskites, we assign the origin of these processes to the AZR^+^ motion, which is to some extent still present in the LT phase. The determined activation energies in the LT phase are very close to the DFT calculated rotation barriers of AZR^+^ cations (Table 2) supporting this assignment. Note that *E*_a_ values of a similar order of magnitude were also obtained for the MA^+^ cation dynamics in the MAPbX_3_ compounds.^3,4,26,29^

The overall temperature dependence of the dielectric permittivity of AZRPbCl_3_ is very similar to the bromide case (Figure 8c,d), except that for this compound, two PTs can be resolved at about 156 and 151 K in agreement with the DSC results. Two dielectric relaxations are also clearly present above and below the PT with the activation energies of 130(5) and 113(5) meV for the higher- and lower-temperature processes, respectively (Figures S16 and S17). The obtained values are similar to the AZRPbBr_3_ case, indicating the same origin of the dipolar dynamics. Note that for this compound we were not able to reliably characterize the dynamics of the higher-temperature dielectric relaxation below the PT point due to a substantial overlap of the processes.

**Figure 9:**
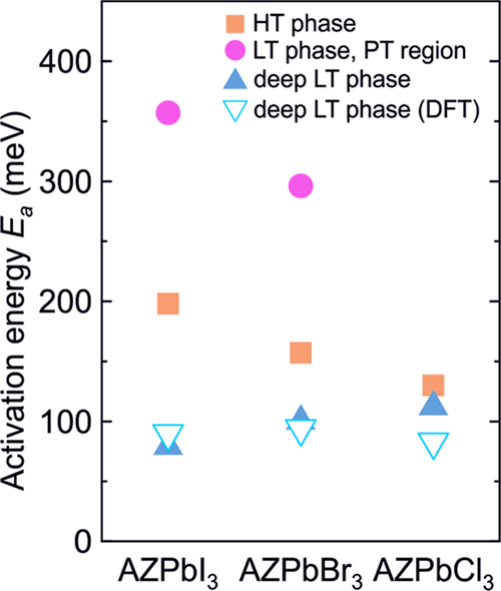
Activation energies of the HT and LT dielectric relaxations of AZRPbX_3_ compounds together with the DFT calculations of the smallest rotational barriers of the AZR^+^ cations.

The temperature-dependent dielectric response of AZRPbI_3_ is also very similar (Figure 8e,f). For this compound, an anomalous decrease of ε′ due to the PT occurs at about 125 K, in good agreement with other experiments. As for the bromide and chloride analogues, two pronounced dielectric relaxations can also be observed for AZRPbI_3_. One relaxation starts above the phase-transition point and crosses the transition, while another one is present below 100 K (Figure 8f). The activation energy of the HT relaxation is 198(5) meV, which roughly doubles as the PT point is crossed (357(5) meV) (see SI for more details; Figures S18 and S19). This behavior is very close to that observed for the bromide analogue. The LT relaxation has an activation energy of 80(5) meV. The obtained values of *E*_a_ for all three compounds are summarized in Figure 9 together with the DFT calculations of the smallest rotational barriers of AZR^+^ cations deep in the LT phase (see Table 2).

Despite powder samples, the measured LT value of the dielectric permittivity remained rather high (∼20), suggesting the lattice polarizability by the lone-pair electrons of the lead cations recently proposed by Fabini et al.^63^ In addition, the high permittivity value is expected to provide efficient screening of the photogenerated carrier and defect states as in the related 3D lead halide perovskites.^29,64^

### NLO Studies

The primary aim of the NLO study was to spectroscopically examine the symmetry of all crystal phases of AZRPbX_3_ perovskites in a broad temperature range, i.e., to see whether any of the described crystal phases generate a second harmonic of radiation, which would indicate its noncentrosymmetric nature. The crystalline powders of the studied perovskites were irradiated with femtosecond laser pulses at selected wavelengths (AZRPbCl_3_―800 nm, AZRPbBr_3_―800 nm, and 1300 nm, and AZRPbI_3_―1300 nm) in a temperature range of 93–293 K upon heating and cooling runs. The wavelengths at which the latter two perovskites were examined were chosen to alleviate the possible self-filtering effect, also referred to as the self-absorption effect,^65^ which is particularly notorious for perovskites due to their high absorption coefficients across the wide spectral range, as found for the MHy family.^6,16^ The temperature-resolved irradiation experiments performed, however, did not show any signatures of the SHG response (see experimental spectra in Figures S20–S27), confirming the centrosymmetric order of these materials across all of the crystal phases.

**Figure 10:**
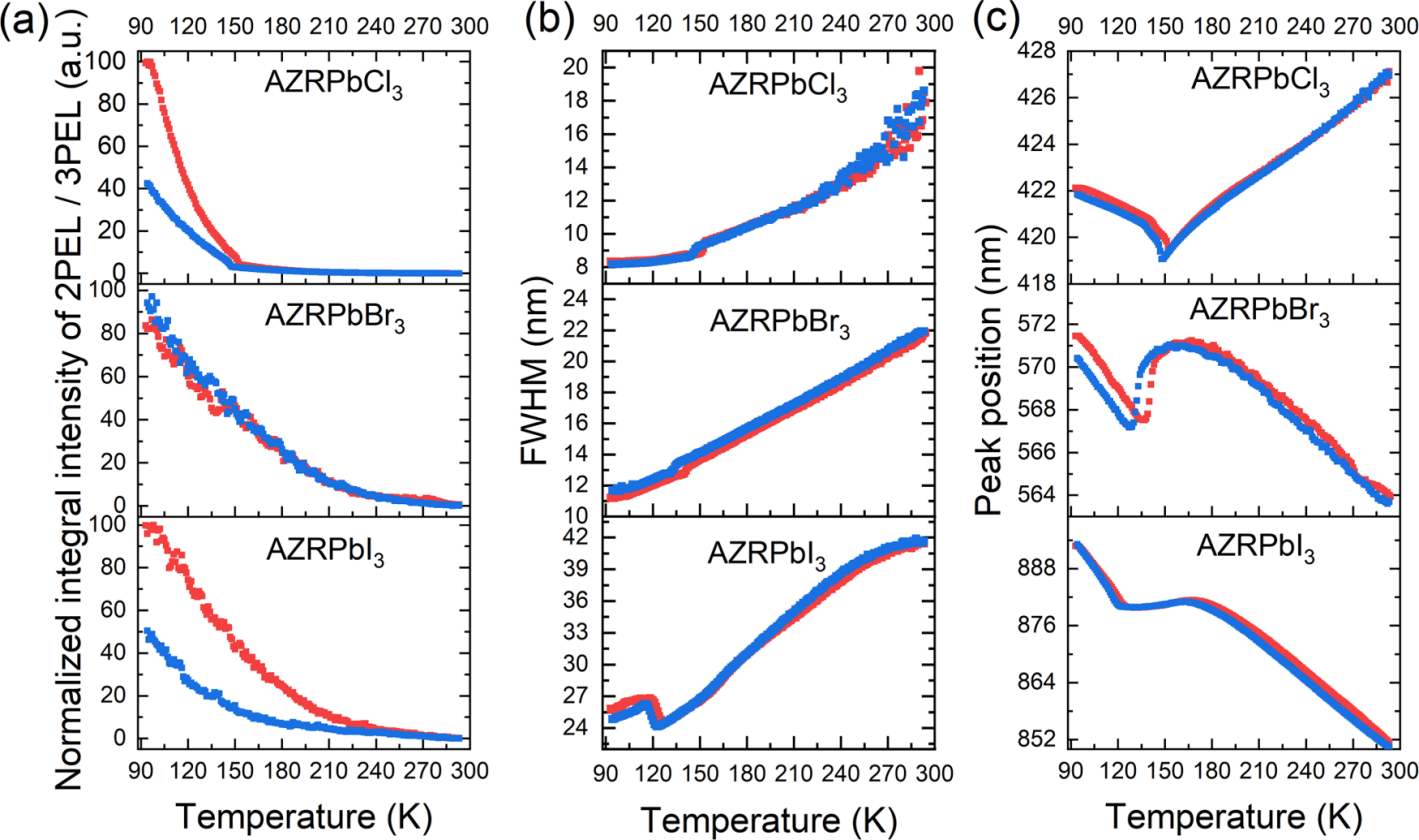
Temperature plots of (a) normalized integral intensities, (b) FWHM, and (c) peak positions of the NLO-induced emissions of AZRPbCl_3_, AZRPbBr_3_, and AZRPbI_3_ excited at 800, 1300, and 1300 nm, respectively.

On the other hand, the collected data revealed intriguing temperature evolution of emission bands reflecting the impact of the structural PTs on the band parameters such as integral intensity, band position, and FWHM. Among the three band parameters, the integral emission intensity seems to be the least sensitive to the crystal phase change (Figure 10a). For example, the temperature plots of integral two-photon excited luminescence (2PEL) for AZRPbBr_3_ and AZRPbI_3_ show an increase of intensity on cooling and no anomalies due to the structural PTs, whereas the AZRPbCl_3_ analogue shows inflection at about 150 K, which is seen in both cooling and heating cycles. In contrast, all temperature plots of line widths (Figure 10b) are clearly indicative of the occurring structural changes, particularly in the case of AZRPbI_3_ for which the signal FWHM broadens about 3 nm, when crossing the PT on cooling; in the case of AZRPbCl_3_ and AZRPbBr_3_, one observes the FWHM narrowing of ca. 2 nm. By far, the strongest effect of the PTs is exerted on the positions of the emission maxima (Figure 10c). Upon cooling from 293 K to the *T*_1_ temperature, the emission of AZRPbBr_3_ shifts to lower energy. Crystal phase change triggers the blue shift of the emission maximum by about 3.7 nm, and further cooling causes a further red shift of emission maxima. Very similar behavior was reported for MAPbBr_3_, which showed ∼4.6 nm blue shift of the one-photon excited emission at the tetragonal to orthorhombic PT.^66^ The same behavior was also reported for the band gap of MAPbBr_3_, which exhibited ∼4 nm blue shift at the tetragonal to orthorhombic PT and two-photon emission of MAPbBr_3_.^67,68^ The red shift in the cubic phase and blue shift at the PT are consistent with the theoretical studies of the iodide analogue, which showed that for the cubic phase the band gap decreases with the decrease of the lattice constant, but it increases with a deviation of I atoms from the cubic symmetry sites.^69^ The red shift of the emission band on cooling is also observed for AZRPbI_3_ (Figure 10c). However, the blue shift is very weak (∼1 nm), and it extends from 164 to 122 K. This behavior suggests that the phase transition of AZRPbI_3_ is associated with smaller deformation of the cubic structure compared to AZRPbBr_3_. Interestingly, an opposite behavior was reported for the MA-based perovskites, i.e., the blue shift at the PT was significantly larger for MAPbI_3_.^25^ An interesting departure from the red shift on cooling in the cubic phase is noted for AZRPbCl_3_, for which the emission exhibits a blue shift on cooling down to the *T*_1_ point followed by the red shift on further cooling in the LT phase. An opposite behavior was reported for the narrow excitonic emission of MAPbCl_3_.^70^

There are two probable reasons the integral intensities are relatively less telling about the presence of the structural PTs. One is that, in general, the thermal quenching itself contributes significantly to the change in the relative intensity of the luminescence band; e.g., upon heating, the luminescence signal intensity drops 2 orders of magnitude or more. From a broad temperature perspective, the effect of the structural PT is effectively overwhelmed by the temperature-dependent drift of the signal intensity. The other reason is the fact that the intensity of the two-photon- and especially multiphoton-induced emissions are superlinearly dependent on the pump intensity. Given the intrinsic fluctuations of the laser source, the obtained integral intensities of 2PEL or three-photon excited luminescence (3PEL) at different temperatures feature an additional signal scatter, which buries signal changes ascribable to the PT. In this context, it is clear why the band position and FWHM were found to be better indicators of the transitions.

Assessment of whether these emissions are due to two- or three-photon absorption could be made only based on the power-dependent measurements. Indeed, to determine the nature of the observed emissions, power-dependent luminescence experiments were conducted at 293 K on powdered samples; the same pumping wavelengths as those for temperature-resolved studies were employed. Figures S28, S30, S32, and S34 present the experimental spectra obtained at different excitation powers, and Figures S29, S31, S33, and S35 show the corresponding log(*I*) = *f*(log(*P*)) plots with the least-squares linear fits for all registered emissions. Based on the obtained slopes, it is apparent that the narrow emissions of AZRPbCl_3_ and AZRPbBr_3_ excited at 800 nm are of the three- and two-photon origin, respectively, and the difference in the mechanism of the nonlinear photon absorption is a direct consequence of the halide-induced difference in optical band gaps between these two materials. Likewise, a very narrow band gap of AZRPbI_3_ perovskite is the primary reason that at 1300 nm the log–log plot produces a slope close to 2, which indicates a two-photon absorption (2PA) at this wavelength. This is in line with the extant few instances of hybrid perovskites, which reflect the immense impact of halide content on the number of photons involved in the nonlinear absorption event. Indeed, in the NIR region, in the vicinity of 1300 nm, a strong 2PA was found for MAPb_0.75_Sn_0.25_I_3_,^71^ whereas the three-photon absorption (3PA) was so far reported for MAPbBr_3_/(OA)_2_PbBr_4_ nanocubes, ZJU-28⊃MAPbBr_3_ hybrid, and (IA)_2_(MA)_2_Pb_3_Br_10_.^72−74^ The comparison of nonlinear absorption parameters is challenging due to different conventions employed for reporting of the NLO parameters, but it should be noted, however, that if the maximization of nonlinear emission intensity is of interest, the two-photon-absorbing iodine-containing perovskites could be preferred. In general, the simultaneous absorption of two photons is a much more likely event than that of three photons or more, which consequently translates to the lower absolute intensity of multiphoton-excited luminescence (MPEL) signals compared to 2PEL.

The SSTPEF technique^75^ was employed to quantify the two-photon brightness, σ_2_φ, of AZRPbBr_3_ (800 nm) and AZRPbI_3_ (1300 nm) at room temperature, with bis(4-diphenylamino)stilbene (BDPAS) and Styryl 9M serving as two-photon reference compounds, see Figures S36 and S37 for experimental spectra.^76^ The results revealed that the value of σ_2_φ at 800 nm is 234 GM per structural unit of AZRPbBr_3_, which is an order of magnitude higher than that for MHy_2_PbBr_4_ (13 GM) at the same wavelength.^6^ In the case of AZRPbI_3_, the value of σ_2_φ was found to be 101 GM at 1300 nm per unit formula. This high two-photon brightness of emission stands out among lead perovskites emitting in the NIR portion of the spectrum,^77^ as well as is of comparable relative strength as two-photon organic dyes are especially optimized to work in the NIR-II spectral region.^78^

## Conclusions

We have conducted a thorough study of the structural, dielectric, phonon, and NLO properties of the novel 3D AZRPbX_3_ perovskites to map the phase diagrams and elucidate the dynamics of AZR^+^ cations as well as the mechanism of the structural PTs in this family of compounds. Understanding all of these aspects is highly important for the applicability of these perovskites in emerging optoelectronic applications.

Our DSC studies revealed that on heating, AZRPbBr_3_ and AZRPbI_3_ exhibit a single PT at 145 and 132 K, respectively, while AZRPbCl_3_ undergoes two PTs at 145 and 154 K. Analysis of the PT entropies showed that these transitions have an order–disorder mechanism, but the contribution of the ordering/disordering processes to the mechanism of the PTs seems to decrease in the order Cl > Br > I.

X-ray diffraction data revealed that the LT polymorphs of AZRPbX_3_ (X = Br, Cl) exhibit crystal structures that differ from the previously reported 3D lead halide perovskites. The LT crystal structure could be solved for AZRPbBr_3_ in the *R*3̅*c* space group symmetry, and it confirmed an order–disorder mechanism of the PT for this compound. In the case of chloride and iodide, fine details of the crystal structures could not be obtained. However, by examining the powder diffraction patterns of AZRPbI_3_, we observed that the structure of this compound corresponds to the primitive P cell of the *Pmna* space group, similar to the LT phases of MAPbI_3_ and CsPbI_3_. In the case of AZRPbCl_3_, the diffraction pattern was compatible with the monoclinic distortion, like in the γ-phase of CsPbCl_3_, for which the monoclinic *P*2_1_/*m* phase was postulated. The characteristic feature of this phase is the presence of two unique sites occupied by Cs^+^ cations.

Raman studies of AZRPbBr_3_ and AZRPbCl_3_ confirmed that the PTs in these compounds are triggered by the ordering of the AZR^+^ cations. They also revealed the presence of two distinct AZR^+^ cations in the LT phase of AZRPbCl_3_ and showed a larger number of bands observed at 80 K for AZRPbCl_3_ compared to AZRPbBr_3_, suggesting lower symmetry of the chloride framework. These features are consistent with the postulated monoclinic distortion of the LT phase of AZRPbCl_3_.

We also employed dielectric spectroscopy to study the dielectric response and cation dynamics in these materials. For all compounds, PTs were observed as pronounced step-like changes in the real part of the dielectric permittivity, typical for a sudden freezing of the organic cation motion. This behavior is consistent with the order–disorder character of the PTs. For all three compounds, we also observed relatively high values of dielectric permittivity, which should provide efficient screening of the photogenerated carriers and defect sites in the photovoltaic devices employing these materials.

The microscopic picture of the PTs and cation dynamics was also studied using atomistic modeling. MD simulations revealed the presence of structural transitions in all three compounds with temperatures close to the experimental values. The calculated rotation barriers of the AZR^+^ cations were found to be in good agreement with the activation energies measured by the dielectric spectroscopy. This provided further support that the dielectric response of these materials is mainly dominated by the dynamics of the AZR^+^ cations.

Our NLO studies revealed an absence of SHG signals for all compounds, proving that all phases of the AZRPbX_3_ perovskites are centrosymmetric. AZRPbCl_3_ showed intense 3PEL, while AZRPbBr_3_ and AZRPbI_3_ exhibited efficient 2PEL. Analysis of the positions of the emission maxima revealed clear anomalies at the PTs and suggested that the PT of AZRPbI_3_ is associated with smaller deformation of the cubic structure compared to AZRPbBr_3_. Our data also showed that AZRPbBr_3_ and AZRPbI_3_ possess very high two-photon brightness, making these perovskites promising for third-order NLO applications.

## Data Availability

The PDynA package developed in this work is open-source and available online at https://github.com/WMD-group/PDynA (DOI: 10.5281/zenodo.7948045).

## Supporting Information Available

Crystal data and Raman wavenumbers (Tables S1–S4); powder diffraction data and Rietveld refinement, relative energy of AZRPbBr_3_ per formula unit, temperature-dependent Raman spectra, frequency dependencies of the dielectric permittivity, Arrhenius plots, experimental upconverted temperature-dependent luminescence spectra upon 800 and 1300 nm excitation, power-dependent spectra, and log–log plot of integral intensities plotted as a function of applied laser (Figures S1–S37) (PDF)

## References

[ref1] Saparov B.; Mitzi D. B. Organic-Inorganic Perovskites: Structural Versatility for Functional Materials Design. Chem. Rev. 2016, 116, 4558–4596. 10.1021/acs.chemrev.5b00715. 27040120

[ref2] Smith M. D.; Connor B. A.; Karunadasa H. I. Tuning the Luminescence of Layered Halide Perovskites. Chem. Rev. 2019, 119, 3104–3139. 10.1021/acs.chemrev.8b00477. 30689364

[ref3] Šimėnas M.; Balciunas S.; Gągor A.; Pieniążek A.; Tolborg K.; Kinka M.; Klimavicius V.; Svirskas S.; Kalendra V.; Ptak M.; et al. Mixology of MA_1–*x*_EA_*x*_PbI_3_ Hybrid Perovskites: Phase Transitions, Cation Dynamics, and Photoluminescence. Chem. Mater. 2022, 34, 10104–10112. 10.1021/acs.chemmater.2c02807. 36439319 PMC9686138

[ref4] Simenas M.; Balciunas S.; Wilson J. N.; Svirskas S.; Kinka M.; Garbaras A.; Kalendra V.; Gagor A.; Szewczyk D.; Sieradzki A.; et al. Suppression of Phase Transitions and Glass Phase Signatures in Mixed Cation Halide Perovskites. Nat. Commun. 2020, 11, 5103 10.1038/s41467-020-18938-z. 33037192 PMC7547736

[ref5] Tu Y.; W J.; Xu G.; Yang X.; Cai R.; Gong Q.; Zhu R.; Huang W. Perovskite Solar Cells for Space Applications: Progress and Challenges. Adv. Mater. 2021, 33, 2006545 10.1002/adma.202006545. 33861877

[ref6] Mączka M.; Zaręba J. K.; Gągor A.; Stefańska D.; Ptak M.; Rolder K.; Kajewski D.; Soszyński A.; Fedoruk K.; Sieradzki A. [Methylhydrazinium]_2_PbBr_4_, a Ferroelectric Hybrid Organic-Inorganic Perovskite with Multiple Nonlinear Optical Outputs. Chem. Mater. 2021, 33, 2331–2342. 10.1021/acs.chemmater.0c04440.

[ref7] Kar S.; Jamaludin N. F.; Yantara N.; Mhaisalkar S. G.; Leong W. L. Recent Advancements and Perspectives on Light Management and High Performance in Perovskite Light-Emitting Diodes. Nanophotonics 2021, 10, 2103–2143. 10.1515/nanoph-2021-0033.

[ref8] Ptak M.; Sieradzki A.; Simenas M.; Mączka M. Molecular Spectroscopy of Hybrid Organic-Inorganic Perovskites and Related Compounds. Coord. Chem. Rev. 2021, 448, 214180 10.1016/j.ccr.2021.214180.

[ref9] Mączka M.; Sobczak S.; Ratajczyk P.; Leite F. F.; Paraguassu W.; Dybała F.; Herman A. P.; Kudrawiec R.; Katrusiak A. Pressure-Driven Phase Transition in Two-Dimensional Perovskite MHy_2_PbBr_4_. Chem. Mater. 2022, 34, 7867–7877. 10.1021/acs.chemmater.2c01533.

[ref10] Weber D. CH_3_NH_3_PbX_3_, Ein Pb(II)-System mit Kubischer Perowskitstruktur/CH_3_NH_3_PbX_3_, a Pb(II)-System with Cubic Perovskite Structure. Z. Naturforsch. B 1978, 33, 1443–1445. 10.1515/znb-1978-1214.

[ref11] Kojima A.; Teshima K.; Shirai Y.; Miyasaka T. Organometal Halide Perovskites as Visible-Light Sensitizers for Photovoltaic Cells. J. Am. Chem. Soc. 2009, 131, 6050–6051. 10.1021/ja809598r. 19366264

[ref12] Stoumpos C. C.; Malliakas C. D.; Kanatzidis M. G. Semiconducting Tin and Lead Iodide Perovskites with Organic Cations: Phase Transitions, High Mobilities, and Near-Infrared Photoluminescent Properties. Inorg. Chem. 2013, 52, 9019–9038. 10.1021/ic401215x. 23834108

[ref13] Alsalloum A. Y.; Turedi B.; Zheng X.; Mitra S.; Zhumekenov A. A.; Lee K. J.; Maity P.; Gereige I.; AlSaggaf A.; Roqan I. S.; et al. Low-temperature Crystallization Enables 21.9% Efficient Single-Crystal MAPbI_3_ Inverted Perovskite Solar Cells. ACS Energy Lett. 2020, 5, 657–662. 10.1021/acsenergylett.9b02787.

[ref14] Mahapatra A.; Prochowicz D.; Kruszyńska J.; Satapathi S.; Akin S.; Kumari H.; Kumar P.; Fazel Z.; Tavakoli M. M.; Yadav P. Effect of Bromine Doping on the Charge Transfer, Ion Migration and Stability of the Single Crystalline MAPb(Br_x_I_1-x_)_3_ Photodetector. J. Mater. Chem. C 2021, 9, 15189–15200. 10.1039/D1TC04208D.

[ref15] Liu H.; Zhang H.; Xu X.; Zhang L. The Opto-Electronic Functional Devices Based on Three-Dimensional Lead Halide Perovskites. Appl. Sci. 2021, 11, 1453 10.3390/app11041453.

[ref16] Ma̧czka M.; Ptak M.; Gągor A.; Stefańska D.; Zaręba J. K.; Sieradzki A. Methylhydrazinium Lead Bromide: Noncentrosymmetric Three-Dimensional Perovskite with Exceptionally Large Framework Distortion and Green Photoluminescence. Chem. Mater. 2020, 32, 1667–1673. 10.1021/acs.chemmater.9b05273.

[ref17] Ma̧czka M.; Gągor A.; Zaręba J. K.; Stefańska D.; Drozd M.; Balciunas S.; Simenas M.; Banys J.; Sieradzki A. Three-Dimensional Perovskite Methylhydrazinium Lead Chloride with Two Polar Phases and Unusual Second-Harmonic Generation Bistability above Room Temperature. Chem. Mater. 2020, 32, 4072–4082. 10.1021/acs.chemmater.0c00973.

[ref18] Drozdowski D.; Gągor A.; Stefańska D.; Zaręba J. K.; Fedoruk K.; Mączka M.; Sieradzki A. Three-Dimensional Methylhydrazinium Lead Halide Perovskites: Structural Changes and Effects on Dielectric, Linear, and Nonlinear Optical Properties Entailed by the Halide Tuning. J. Phys. Chem. C 2022, 126, 1600–1610. 10.1021/acs.jpcc.1c07911.

[ref19] Huang X.; Li X.; Tao Y.; Guo S.; Gu J.; Hong H.; Yao Y.; Guan Y.; Gao Y.; Li C.; et al. Understanding Electron-Phonon Interactions in 3D Lead Halide Perovskites from the Stereochemical Expression of 6s^2^ Lone Pairs. J. Am. Chem. Soc. 2022, 144, 12247–12260. 10.1021/jacs.2c03443. 35767659

[ref20] Zheng C.; Rubel O. Aziridinium Lead Iodide: A Stable, Low-Band-Gap Hybrid Halide Perovskite for Photovoltaics. J. Phys. Chem. Lett. 2018, 9, 874–880. 10.1021/acs.jpclett.7b03114. 29390607

[ref21] Petrosova H. R.; Kucheriv O. I.; Shova S.; Gural’skiy I. A. Aziridinium Cation Templating 3D Lead Halide Hybrid Perovskites. Chem. Commun. 2022, 58, 5745–5748. 10.1039/D2CC01364A. 35446324

[ref22] Stefańska D.; Ptak M.; Mączka M. Synthesis, Photoluminescence and Vibrational Properties of Aziridinium Lead Halide Perovskites. Molecules 2022, 27, 7949 10.3390/molecules27227949. 36432050 PMC9698367

[ref23] Semenikhin O. A.; Kucheriv O. I.; Sacarescu L.; Shova S.; Gural’skiy I. A. Quantum Dots Assembled from an Aziridinium Based Hybrid Perovskite Displaying Tunable Luminescence. Chem. Commun. 2023, 59, 3566–3569. 10.1039/D2CC06791A. 36880308

[ref24] Dar M. I.; Jacopin G.; Meloni S.; Mattoni A.; Arora N.; Boziki A.; Zakerruddin S. M.; Rothlisberger U.; Grätzel M. Origin of Unusual Bandgap Shift and Dual Emission in Organic-Inorganic Lead Halide Perovskites. Sci. Adv. 2016, 2, e1601156 10.1126/sciadv.1601156. 27819049 PMC5091363

[ref25] Wright A. D.; Verdi C.; Milot R. L.; Eperon G. E.; Pérez-Osorio M. A.; Snaith H. J.; Giustino F.; Johnston M. B.; Herz M. L. Electron-Phonon Coupling in Hybrid Lead Halide Perovskites. Nat. Commun. 2016, 7, 11755 10.1038/ncomms11755. PMC4894981 27225329

[ref26] Šimėnas M.; Balciunas S.; Svirskas S.; Kinka M.; Ptak M.; Kalendra V.; Gagor A.; Szewczyk D.; Sieradzki A.; Grigalaitis R.; et al. Phase Diagram and Cation Dynamics of Mixed MA_1-x_FA_x_PbBr_3_ Hybrid Perovskites. Chem. Mater. 2021, 33, 5926–5934. 10.1021/acs.chemmater.1c00885.

[ref27] Mączka M.; Ptak M. Temperature-Dependent Raman Studies of FAPbBr_3_ and MAPbBr_3_ Perovskites: Effect of Phase Transitions on Molecular Dynamics and Lattice Distortion. Solids 2022, 3, 111–121. 10.3390/solids3010008.

[ref28] Mączka M.; Zienkiewicz J. A.; Ptak M. Comparative Studies of Phonon Properties of Three-Dimensional Hybrid Organic-Inorganic Perovskites Comprising Methylhydrazinium, Methylammonium, and Formamidinium Cations. J. Phys. Chem. C 2022, 126, 4048–4056. 10.1021/acs.jpcc.1c09671.

[ref29] Anusca I.; Balciunas S.; Gemeiner P.; Svirskas S.; Sanlialp M.; Lackner G.; Fettkenhauer C.; Belovickis J.; Samulionis V.; Ivanov M.; et al. Dielectric Response: Answer to Many Questions in the Methylammonium Lead Halide Solar Cell Absorbers. Adv. Energy Mater. 2017, 7, 1700600 10.1002/aenm.201700600.

[ref30] Whitfield P. S.; Herron N.; Guise W. E.; Page K.; Cheng Y. Q.; Milas I.; Crawford M. K. Structures, Phase Transitions and Tricritical Behavior of the Hybrid Perovskite Methyl Ammonium Lead Iodide. Sci. Rep. 2016, 6, 35685 10.1038/srep35685. 27767049 PMC5073364

[ref31] Oku T. Crystal Structures of Perovskite Halide Compounds Used for Solar Cells. Rev. Adv. Mater. Sci. 2020, 59, 264–305. 10.1515/rams-2020-0015.

[ref32] Mozur E. M.; Trowbridge J. C.; Maughan A. E.; Gorman M. J.; Brown C. M.; Prisk T. R.; Neilson J. R. Dynamical Phase Transitions and Cation Orientation-Dependent Photoconductivity in CH(NH_2_)_2_PbBr_3_. ACS Mater. Lett. 2019, 1, 260–264. 10.1021/acsmaterialslett.9b00209.

[ref33] Mączka M.; Ptak M.; Vasconcelos D. L. M.; Giriunas L.; Freire P. T. C.; Bertmer M.; Banys J.; Simenas M. NMR and Raman Scattering Studies of Temperature-and Pressure-Driven Phase Transitions in CH_3_NH_2_NH_2_PbCl_3_ Perovskite. J. Phys. Chem. C 2020, 124, 26999–27008. 10.1021/acs.jpcc.0c07886.

[ref34] Mohanty A.; Swain D.; Govinda S.; Row T. N. G.; Sarma D. D. Phase Diagram and Dielectric Properties of MA_1-x_FA_x_PbI_3_. ACS Energy Lett. 2019, 4, 2045–2051. 10.1021/acsenergylett.9b01291.

[ref35] Govinda S.; Kore B. P.; Swain D.; Hossain A.; De C.; Row T. N. G.; Sarma D. D. Critical Comparison of FAPbX_3_ and MAPbX_3_ (X = Br and Cl): How Do They Differ?. J. Phys. Chem. C 2018, 122, 13758–13766. 10.1021/acs.jpcc.8b00602.

[ref36] Petricek V.; Dusek M.; Palatinus L. Crystallographic Computing System JANA2006: General Features. Z. Kristallogr. 2014, 229, 345–352. 10.1515/zkri-2014-1737.

[ref37] Dolomanov O. V.; Bourhis L. J.; Gildea R. J.; Howard J. A. K.; Puschmann K. OLEX2: a Complete Structure Solution, Refinement and Analysis Program. J. Appl. Crystallogr. 2009, 42, 339–341. 10.1107/S0021889808042726.

[ref38] Sheldrick G. M. Crystal Structure Refinement with SHELXL. Acta Crystallogr., Sect. C: Struct. Chem. 2015, 71, 3–8. 10.1107/S2053229614024218. 25567568 PMC4294323

[ref39] Clark R. C.; Reid J. S. The Analytical Calculation of Absorption in Multifaceted Crystals. Acta Crystallogr., Sect. A: Found. Crystallogr. 1995, 51, 887–897. 10.1107/S0108767395007367.

[ref40] Jinnouchi R.; Miwa K.; Karsai F.; Kresse G.; Asahi R. On-the-Fly Active Learning of Interatomic Potentials for Large-Scale Atomistic Simulations. J. Phys. Chem. Lett. 2020, 11, 6946–6955. 10.1021/acs.jpclett.0c01061. 32787192

[ref41] Kresse G.; Furthmüller F. Efficiency of Ab-Initio Total Energy Calculations for Metals and Semiconductors Using a Plane-Wave Basis Set. Comput. Mater. Sci. 1996, 6, 15–50. 10.1016/0927-0256(96)00008-0.

[ref42] Kresse G.; Furthmüller F. Efficient Iterative Schemes for Ab Intio Total-Energy Calculations Using a Plane-Wave Basis Set. Phys. Rev. B 1996, 54, 11169 10.1103/PhysRevB.54.11169. 9984901

[ref43] Liang X.; Klarbring J.; Baldwin W.; Li Z.; Csanyi G.; Walsh A. Structural Dynamics Descriptors for Metal Halide Perovskites. J. Phys. Chem. C 2023, 127 (38), 19141–19151. 10.1021/acs.jpcc.3c03377. PMC10544022 37791100

[ref44] Perdew J. P.; Ruzsinszky A.; Csonka G. I.; Vydrov O. A.; Scuseria G. E.; Constantin L. A.; Zhou X.; Burke K. Restoring the Density-Gradient Expansion for Exchange in Solids and Surfaces. Phys. Rev. Lett. 2008, 100, 136406 10.1103/PhysRevLett.100.136406. 18517979

[ref45] Onoda-Yamamuro N.; Matsuo T.; Suga H. Calorimetric and IR Spectroscopic Studies of Phase Transitions in Methylammonium Trihalogenoplumbates (II). J. Phys. Chem. Solids 1990, 51, 1383–1395. 10.1016/0022-3697(90)90021-7.

[ref46] Poglitsch A.; Weber D. Dynamic Disorder in methylammoniumtrihalogenoplumbates (II) Observed by Millimeter-Wave Spectroscopy. J. Chem. Phys. 1987, 87, 6373–6378. 10.1063/1.453467.

[ref47] Sharma V. K.; Mukhopadhyay R.; Mohanty A.; Sakai V. G.; Tyagi M.; Sarma D. D. Influence of the Halide Ion on the A-site Dynamics in FAPbX_3_ (X = Br and Cl). J. Phys. Chem. C 2022, 126, 7158–7168. 10.1021/acs.jpcc.2c00968.

[ref48] Yang B.; Ming W.; Du M. H.; Keum J. K.; Puretzky A. A.; Rouleau C. M.; Huang J.; Geohegan D. B.; Wang X.; Xiao K. Real-Time Observation of Order-Disorder Transformation of Organic Cations Induced Phase Transition and Anomalous Photoluminescence in Hybrid Perovskites. Adv. Mater. 2018, 30, 1705801 10.1002/adma.201705801. 29660765

[ref49] Fabini D. H.; Stoumpos C. C.; Laurita G.; Kaltzoglou A.; Kontos A. G.; Falaras P.; Kanatzidis M. G.; Seshadri R. Reentrant Structural and Optical Properties and Large Positive Thermal Expansion in Perovskite Formamidinium Lead Iodide. Angew. Chem., Int. Ed. 2016, 55, 15392–15396. 10.1002/anie.201609538. 27862778

[ref50] Weller M. T.; Weber O. J.; Henry P. F.; Di Pumpo A. M.; Hansen T. C. Complete Structure and Cation Orientation in the Perovskite Photovoltaic Methylammonium Lead Iodide Between 100 and 352 K. Chem. Commun. 2015, 51, 4180–4183. 10.1039/C4CC09944C. 25634426

[ref51] Whitfield P. S.; Herron N.; Guise W. E.; Page K.; Cheng Y. Q.; Milas I.; Crawford M. K. Structure, Phase Transitions and Tricritical Behavior of the Hybrid Perovskite Methyl Ammonium Lead Iodide. Sci. Rep. 2016, 6, 35685 10.1038/srep35685. 27767049 PMC5073364

[ref52] Howard C. J.; Kennedy B. J.; Woodward P. M. Ordered Double Perovskites - a Group-Theoretical Analysis. Acta Crystallogr., Sect. B: Struct. Sci. 2003, 59, 463–471. 10.1107/S0108768103010073. 12947230

[ref53] Klarbring J. Low-Energy Paths for Octahedral Tilting in Inorganic Halide Perovskites. Phys. Rev. B 2019, 99, 104105 10.1103/PhysRevB.99.104105.

[ref54] Chi L.; Swainson I.; Cranswick L.; Her J. H.; Stephens P.; Knop O. The Ordered Phase of Methylammonium Lead Chloride CH_3_ND_3_PbCl_3_. J. Solid State Chem. 2005, 178, 1376–1385. 10.1016/j.jssc.2004.12.037.

[ref55] Kawamura Y.; Mashiyama H. Modulated Structure in Phase II of CH_3_NH_3_PbCl_3_. J. Korean Phys. Soc. 1999, 35, S1437–S1440.

[ref56] Linaburg M. R.; McClure M. T.; Majher J. D.; Woodward P. M. Cs_1–x_Rb_x_PbCl_3_ and Cs_1–x_Rb_x_PbBr_3_ Solid Solutions: Understanding Octahedral Tilting in Lead Halide Perovskites. Chem. Mater. 2017, 29, 3507–3514. 10.1021/acs.chemmater.6b05372.

[ref57] Zhang L.; Wang L.; Wang K.; Zou B. Pressure-Induced Structural Evolution and Optical Properties of Metal-Halide Perovskite CsPbCl_3_. J. Phys. Chem. C 2018, 122, 15220–15225. 10.1021/acs.jpcc.8b05397.

[ref58] Fujii Y.; Hoshino S.; Yamada Y.; Shirane G. Neutron-Scattering Study on Phase Transitions of CsPbCl_3_. Phys. Rev. B 1974, 9, 4549–4559. 10.1103/PhysRevB.9.4549.

[ref59] Baldwin W.; Liang X.; Klarbring J.; Dubajic M.; Dell’Angelo D.; Sulton C.; Caddeo C.; Stransk S. D.; Maltoni A.; Walsh A.; et al. Dynamic Local Structure in Caesium Lead Iodide: Spatial Correlation and Transient Domains. Small 2023, 2303565 10.1002/smll.202303565. 37736694

[ref60] Nakada K.; Matsumoto Y.; Shimoi Y.; Yamada K.; Furukawa Y. Temperature-Dependent Evolution of Raman Spectra of Methylammonium Lead Halide Perovskites, CH_3_NH_3_PbX_3_ (X = I, Br). Molecules 2019, 24, 626 10.3390/molecules24030626. 30754650 PMC6384565

[ref61] Leguy A. M. A.; Goñi A. R.; Frost J. M.; Skelton J.; Brivio F.; Rodríguez-Martínez X.; Weber O. J.; Pallipurath A.; Alonso M. I.; Campoy-Quiles M.; et al. Dynamic Disorder, Phonon Lifetimes, and the Assignment of Modes to the Vibrational Spectra of Methylammonium Lead Halide Perovskites. Phys. Chem. Chem. Phys. 2016, 18, 27051–27066. 10.1039/C6CP03474H. 27346792

[ref62] Frost J. M.; Walsh A. What is Moving in Hybrid Halide Perovskite Solar Cells?. Acc. Chem. Res. 2016, 49, 528–535. 10.1021/acs.accounts.5b00431. 26859250 PMC4794704

[ref63] Fabini D. H.; Seshadri R.; Kanatzidis M. G. The Underappreciated Lone Pair in Halide Perovskites Underpins Their Unusual Properties. MRS Bull. 2020, 45, 467–477. 10.1557/mrs.2020.142.

[ref64] Svirskas Š.; Balciunas S.; Simenas M.; Usevicius G.; Kinka M.; Velicka M.; Kubicki D.; Castillo M. E.; Karabanov A.; Shvartsman V. V.; et al. Phase Transitions, Screening and Dielectric Response of CsPbBr_3_. J. Mater. Chem. A 2020, 8, 14015–14022. 10.1039/D0TA04155F.

[ref65] Zaręba J. K.; Białek M. J.; Janczak J.; Nyk M.; Zoń J.; Samoć M. Beyond Single-Wavelength SHG Measurements: Spectrally-Resolved SHG Studies of Tetraphosphonate Ester Coordination Polymers. Inorg. Chem. 2015, 54, 10568–10575. 10.1021/acs.inorgchem.5b01939. 26491884

[ref66] Chen C.; Hu X.; Lu W.; Chang S.; Shi L.; Li L.; Zhong H.; Han J. B. Elucidating the Phase Transitions and Temperature-Dependent Photoluminescence of MAPbBr_3_ Single Crystal. J. Phys. D.: Appl. Phys. 2018, 51, 045105 10.1088/1361-6463/aaa0ed.

[ref67] Park S.; Seo Y. S.; Ahn C. W.; Woo W. S.; Kyhm J.; Lee S. A.; Kim I. W.; Hwang J. Temperature-Dependent Optical Properties of Hybrid Organic-Inorganic Perovskite Single Crystals (CH_3_NH_3_PbI_3_ and CH_3_NH_3_PbBr_3_). J. Phys. D.: Appl. Phys. 2019, 52, 335302 10.1088/1361-6463/ab20fa.

[ref68] Linnenbank H.; Saliba M.; Gui L.; Metzger B.; Tikhodeev S. G.; Kadro J.; Nasti G.; Abate A.; Hagfeldt A.; Graetzel M.; Giessen H. Temperature-Dependent Two-Photon Photoluminescence of CH_3_NH_3_PbBr_3_: Structural Phase and Exciton to Free Carrier Transition. Opt. Mater. Express 2018, 8, 511–521. 10.1364/OME.8.000511.

[ref69] Kim J.; Lee S. C.; Lee S. H.; Hong K. H. Importance of Orbital Interactions in Determining Electronic Band Structures of Organo-Lead Iodide. J. Phys. Chem. C 2015, 119, 4627–4634. 10.1021/jp5126365.

[ref70] Nandi P.; Giri C.; Swain D.; Manju U.; Topwal D. Room Temperature Growth of CH_3_NH_3_PbCl_3_ Single Crystals by Solvent Evaporation Method. CrysEngComm 2019, 21, 656–661. 10.1039/C8CE01939H.

[ref71] Xie Y.; Fan J.; Liu C.; Chi S.; Wang Z.; Yu H.; Zhang H.; Mai Y.; Wang J. Giant Two-Photon Absorption in Mixed halide Perovskite CH_3_NH_3_Pb_0.75_Sn_0.25_I_3_ Thin Films and Application to Photodetection at Optical Communication Wavelengths. Adv. Opt. Mater. 2018, 6, 1700819 10.1002/adom.201700819.

[ref72] Chen W.; Bhaumik S.; Veldhuis S. A.; Xing G.; Xu Q.; Grätzel M.; Grätzel M.; Mhaisalkar S.; Mhaisalkar S.; Mathews N.; Mathews N.; Sum T. C. Giant Five-Photon Absorption from Multidimensional Core-Shell Halide Perovskite Colloidal Nanocrystals. Nat. Commun. 2017, 8, 15198 10.1038/ncomms15198. 28497780 PMC5437305

[ref73] He H.; Cui Y.; Li B.; Wang B.; Jin C.; Yu J.; Yao L.; Yang Y.; Chen B.; Qian G. Confinement of Perovskite-QDs within a Single MOF Crystal for Significantly Enhanced Multiphoton Excited Luminescence. Adv. Mater. 2019, 31, 1806897 10.1002/adma.201806897. 30549115

[ref74] Li M.; Xu Y.; Han S.; Xu J.; Xie Z.; Liu Y.; Xu Z.; Hong M.; Luo J.; Sun Z. Giant and Broadband Multiphoton Absorption Nonlinearities of a 2D Organometallic Perovskite Ferroelectric. Adv. Mater. 2020, 32, 2002972 10.1002/adma.202002972. 32705717

[ref75] Medishetty R.; Zaręba J. K.; Mayer D.; Samoć M.; Fischer R. A. Nonlinear Optical Properties, Upconversion and Lasing in Metal-Organic Frameworks. Chem. Soc. Rev. 2017, 46, 4976–5004. 10.1039/C7CS00162B. 28621347

[ref76] Makarov N. S.; Drobizhev M.; Rebane A. Two-Photon Absorption Standards in the 550–1600 nm Excitation Wavelength Range. Opt. Express 2008, 16, 4029–4047. 10.1364/OE.16.004029. 18542501

[ref77] Zaręba J. K.; Nyk M.; Samoć M. Nonlinear Optical Properties of Emerging Nano- and Microcrystalline Materials. Adv. Opt. Mater. 2021, 9, 2100216 10.1002/adom.202100216.

[ref78] Shaw P. A.; Forsyth E.; Haseeb F.; Yang S.; Bradley M.; Klausen M. Two-Photon Absorption: An Open Door to the NIR-II Biological Window?. Front. Chem. 2022, 10, 921354 10.3389/fchem.2022.921354. 35815206 PMC9263132

