# Supplementary material for: Phase Transitions, Dielectric Response, and Nonlinear Optical Properties of Aziridinium Lead Halide Perovskites
Source: Chem Mater. 2023 Nov 14;35(22):9725–38. doi: 10.1021/acs.chemmater.3c02200 (PMC10687860; doi:10.1021/acs.chemmater.3c02200)
Supplement: Supplementary file 1 — cm3c02200_si_001.pdf [file cm3c02200_si_001.pdf]

Supporting Information for

Phase Transitions, Dielectric Response and Nonlinear Optical Properties of Aziridinium Lead Halide Perovskites

Mirosław Mączka,[a] Maciej Ptak,[a] Anna Gągor,[a] Jan K. Zaręba,[b] Xia Liang,[c] Sergejus Balčiūnas,[d] Oleksandr A. Semenikhin,[e] Olesia I. Kucheriv,[e] Il'ya A. Gural'skiy,[e] Sergiu Shova,[f] Aron Walsh,[c,g] Jūras Banys,[d] and Mantas Šimėnas[d]*

[a]Institute of Low Temperature and Structure Research, Polish Academy of Sciences, ul. Okólna 2, 50-422 Wrocław, Poland

[b]Institute of Advanced Materials, Faculty of Chemistry, Wrocław University of Science and Technology, 50-370 Wrocław, Poland

[c]Department of Materials, Imperial College London, South Kensington Campus, London SW7 2AZ, UK

[d]Faculty of Physics, Vilnius University, LT-10257 Vilnius, Lithuania

[e]Department of Chemistry, Taras Shevchenko National University of Kyiv, 64 Volodymyrska St., Kyiv 01601, Ukraine

[f]*Department of Inorganic Polymers, Petru Poni Institute of Macromolecular Chemistry, Aleea Grigore Ghica Voda 41-A, Iasi, 700487, Romania*

[g]*Department of Physics, Ewha Womans University, Seoul 03760, Korea*

*Email: m.maczka@intibs.pl

X-ray diffraction

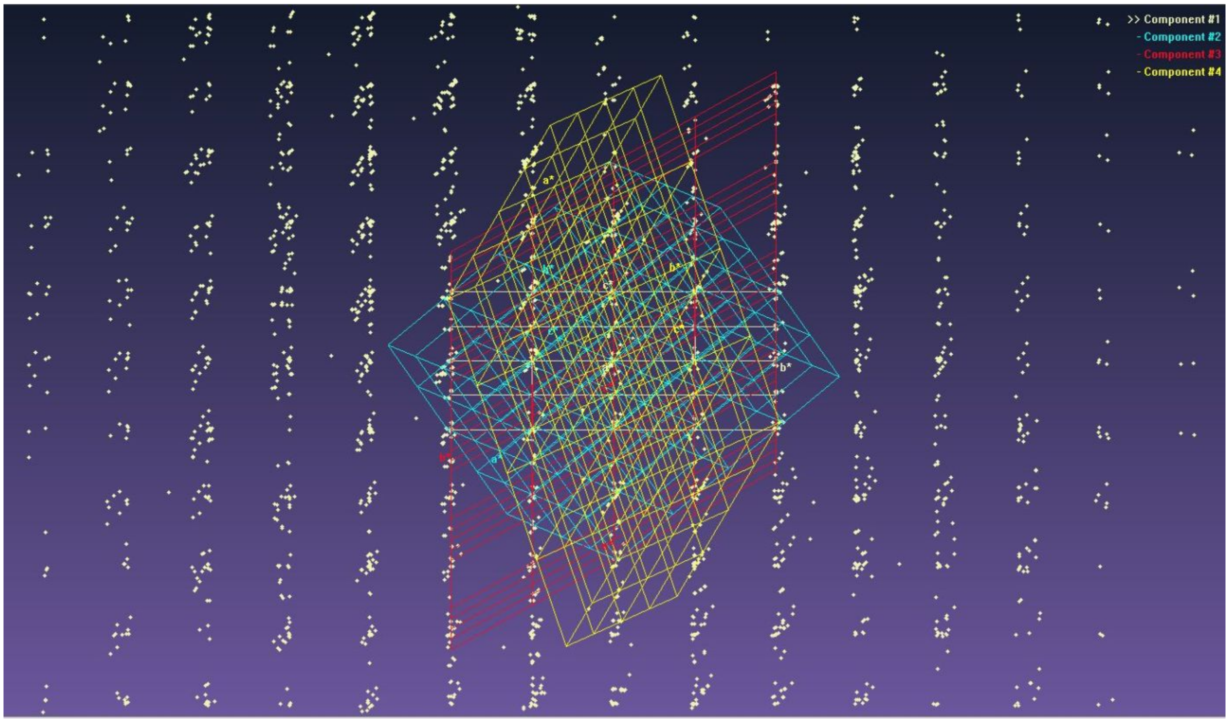

Figure S1. Orthogonal projection (*a*-axis) of a diffraction pattern in reciprocal space ($AZRPbBr_3$).

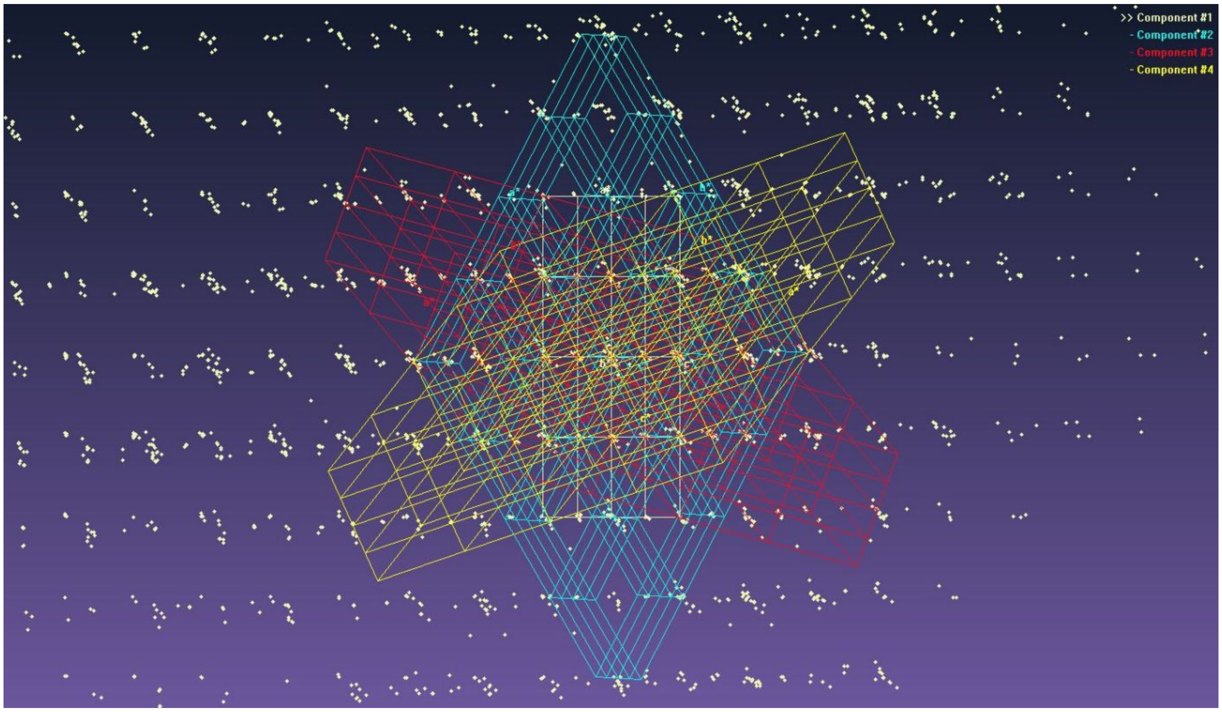

Figure S2. Orthogonal projection (*b*-axis) of a diffraction pattern in reciprocal space ($AZRPbBr_3$).

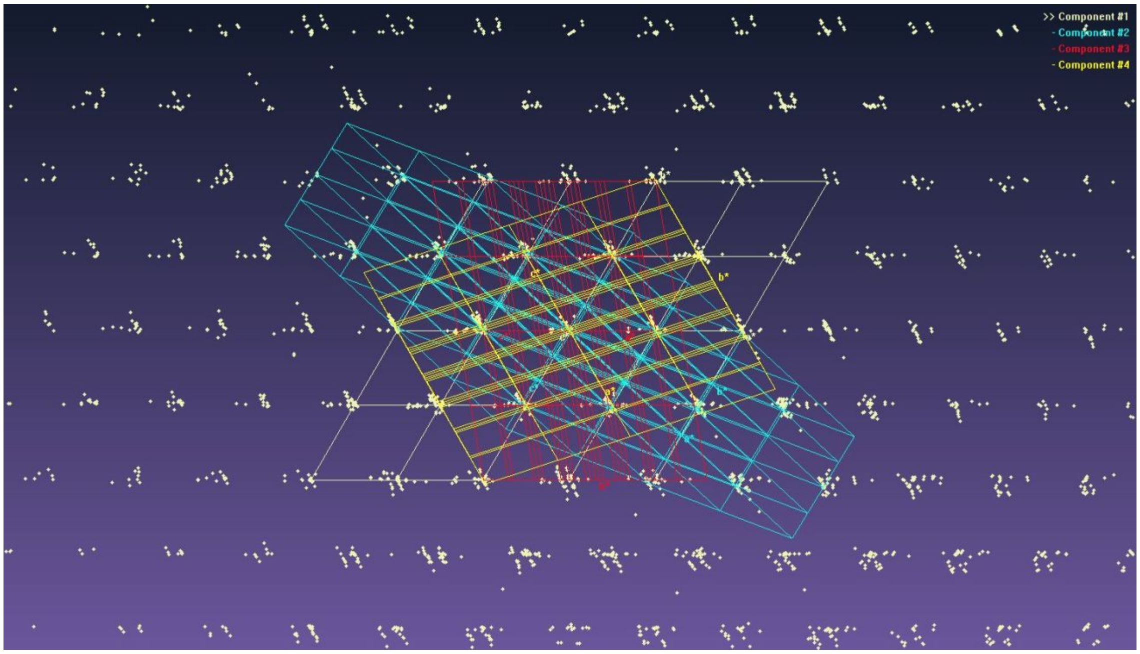

Figure S3. Orthogonal projection (*c*-axis) of a diffraction pattern in reciprocal space ($AZRPbBr_3$).

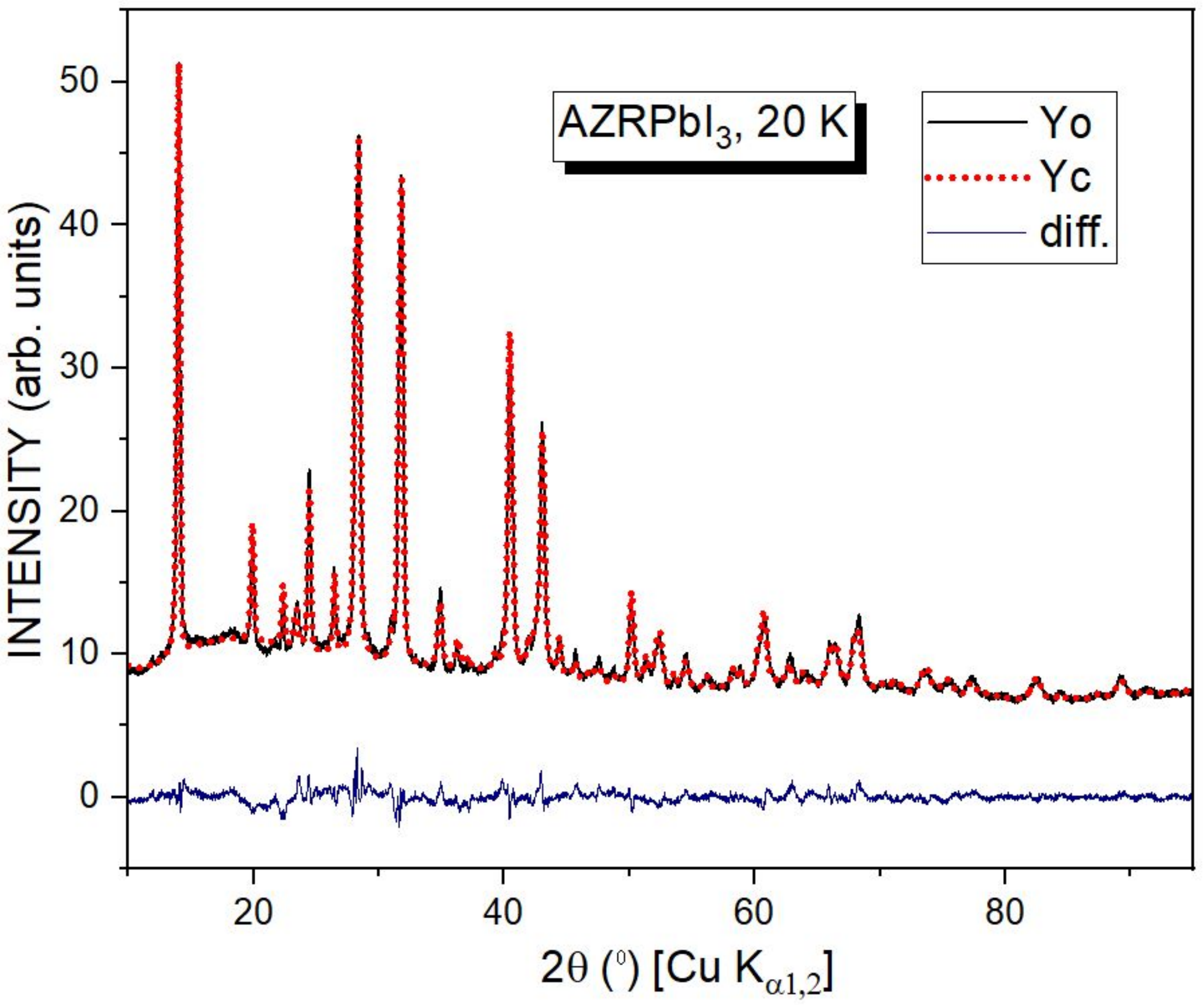

Figure S4. The results of the Rietveld refinement for powder diffraction data of AZRPbI$_3$, *T* =20 K. Crystal system: orthorhombic, space group *Pnma*, lattice parameters a = 8.878(1) Ȧ, b= 12.657 (1) Ȧ and c = 8.859(1) Ȧ, R_{all}= 0.06; wR_{all} = 0.10; GOF = 11.58; R_p = 0.028, wR_p = 0.037.

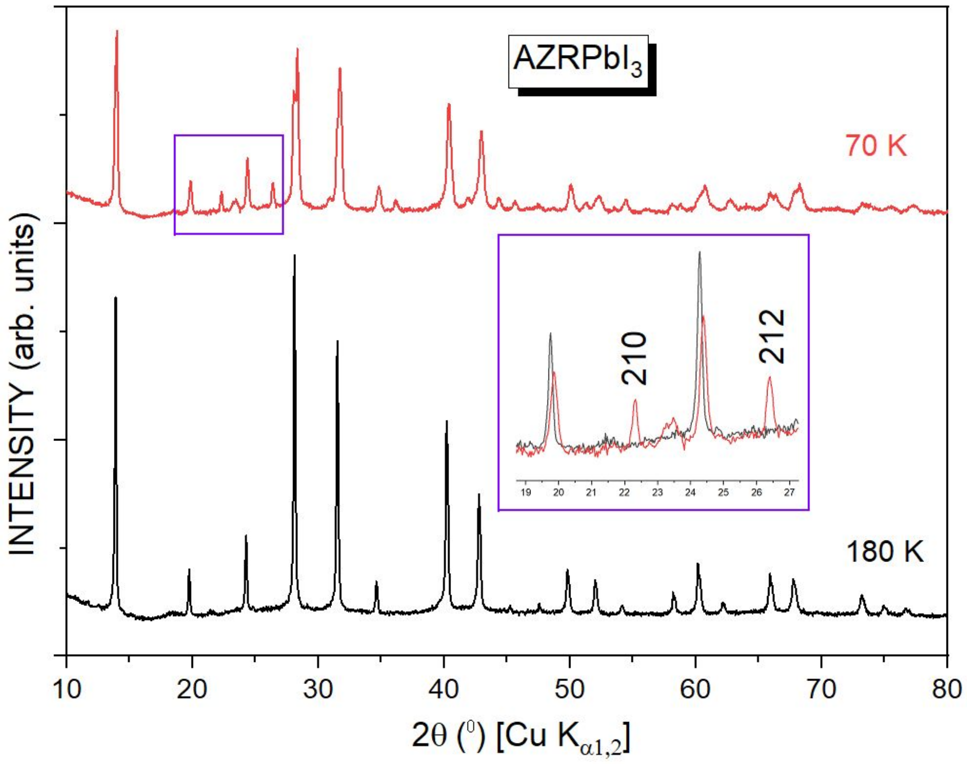

Figure S5. Powder diffraction patterns of the cubic (180 K) and orthorhombic Pnma (70 K) phases of $AZRPbI_3$. The reduction of symmetry manifests as a splitting of diffraction peaks and reduction of their intensity. The 210 and 212 peaks violating reflection conditions for the *I*-cantered lattice (h+k+l=2n) are highlighted in the inset.

The degree of orthorhombic distortion is small, thus, basing on the atomic positions the tetragonal phase is postulated by Platon (see the check cif report). However, due to the presence of Bragg peaks breaking the I cell cantering, the tetragonal *I*4/*mcm* model (LT phase of $MAPbI_3$) was excluded. Also the tetragonal *P*4/*mmm* model of the second LT phase of $MAPbI_3$ does not fit the X-ray data as the unit cell of this phase has reduced volume.

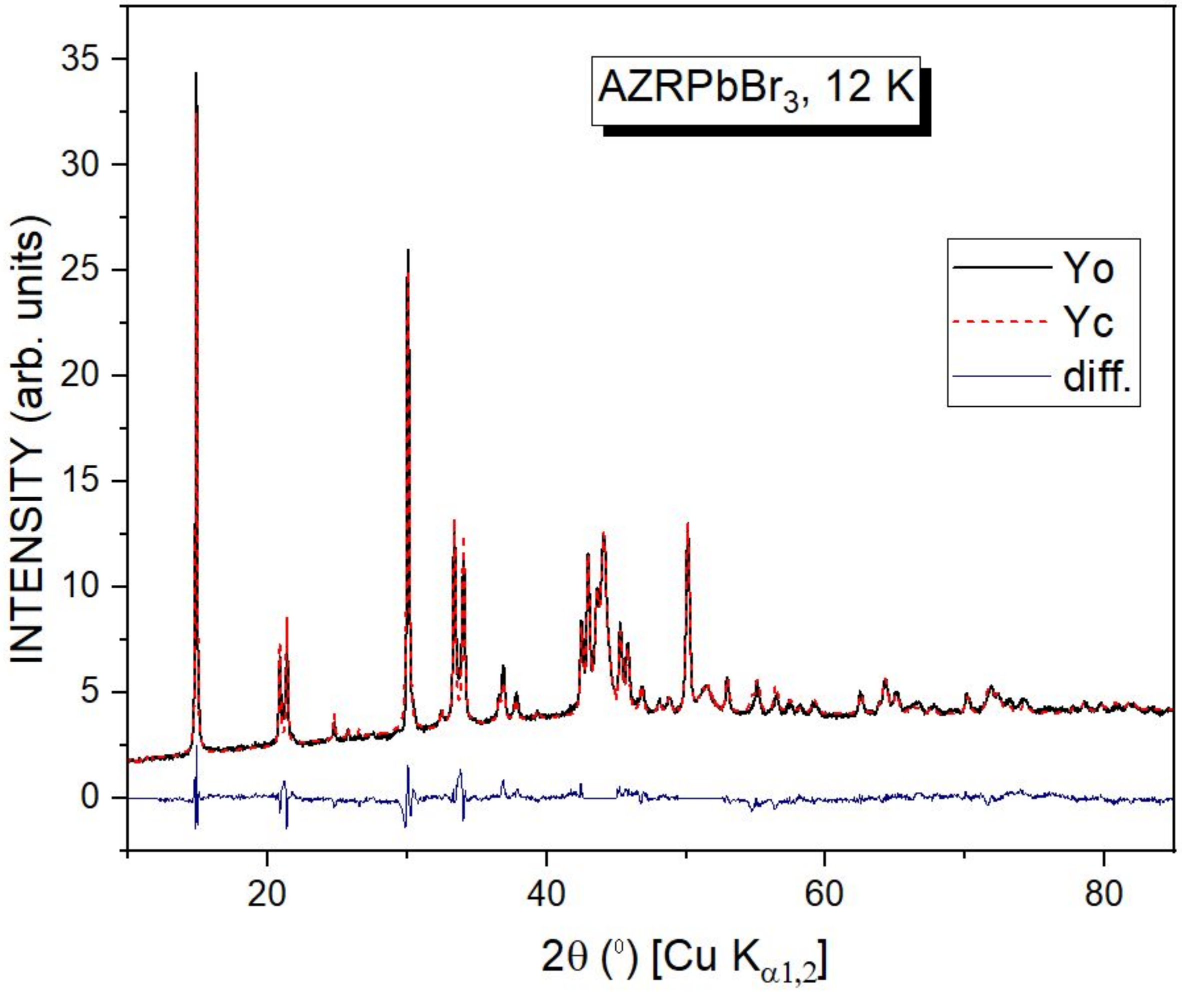

Figure S6. The results of the Rietveld refinement for the powder diffraction data of $AZRPbBr_3$, T = 12 K. Crystal system: trigonal, space group $R\overline{3}c$, lattice parameters a = 8.4974(9) Å, c = 20.105(3) Å, γ = 120° R_{all}=0.125; wR_{all} = 0.114; GOF = 3.01; R_p = 0.032, wR_p = 0.047. The regions marked on the difference intensity as plain lines were excluded from refinement as the intensities were disturbed by the peaks from the holder (~44° and ~50°).

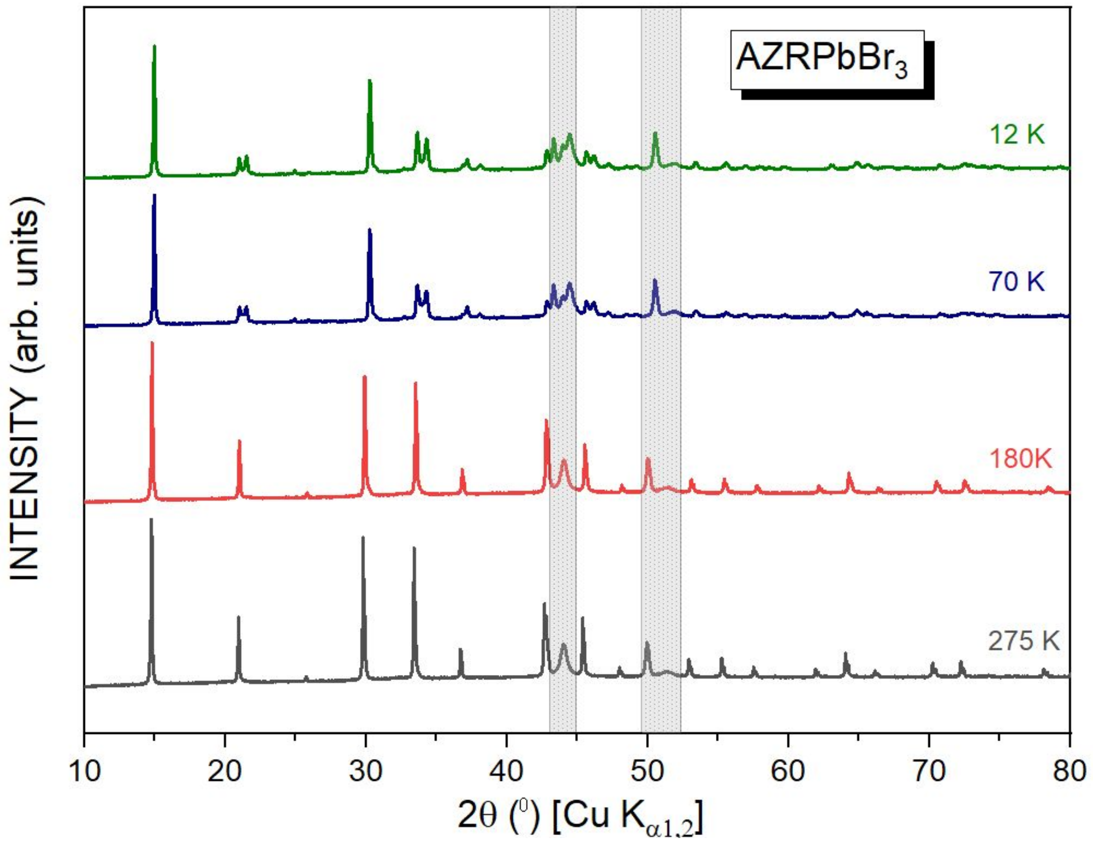

Figure S7. Temperature dependent powder X-ray diffractograms showing the symmetry breaking at low temperatures of $AZRPbBr_3$. The grey areas mark the 2θ thresholds, where the peaks from the sample holder were recorded.

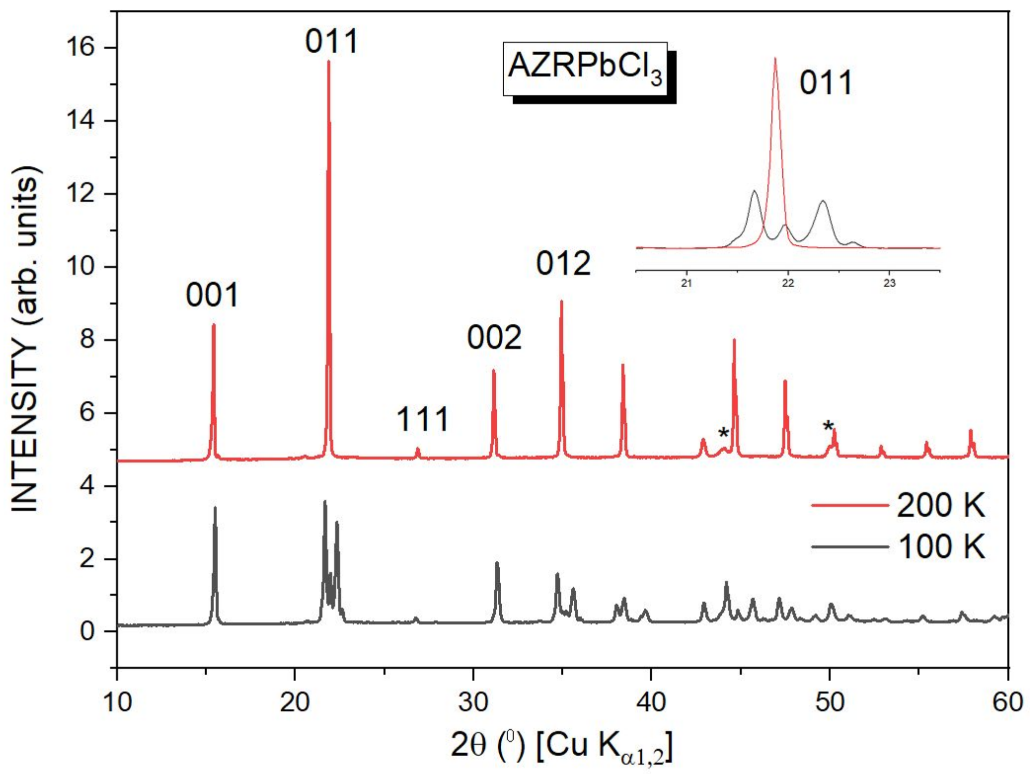

Figure S8. The powder X-ray diffractograms of $AZRPbCl_3$ show the substantial symmetry lowering at low temperature. The peaks at 200 K are indexed in the cubic unit cell of $Pm\overline{3}m$.

Table S1. Crystal data and structure refinement for $AZRPbBr_3$.

Empirical formula	$C_2H_6Br_3NPb$
Formula weight	490.98
Temperature/K	100
Crystal system	trigonal
Space group	$R\overline{3}c$
a/Å	8.4943(13)
c/Å	20.074(4)
γ/°	120
Volume/Å^3	1254.4(5)
Z	6
ρ_{calc}g/cm^3	3.900
μ/mm^{-1}	34.414
Crystal size/mm^3	0.126 × 0.107 × 0.08
2Θ range for data collection/°	6.868 to 58.822
Reflections collected	2726
Independent reflections	2726 [R_{sigma} = 0.0709]
Data/restraints/parameters	2726/0/20
Goodness-of-fit on F^2	1.077
Final *R* indexes [I>=2σ (I)]	R_1 = 0.0494, wR_2 = 0.1394
Final *R* indexes [all data]	R_1 = 0.0642, wR_2 = 0.1451

Table S2. Bond Lengths for $AZRPbBr_3$.

	**Length (Å)**
Pb1– Br1	3.013(12)
Pb1– Br1[3]	2.969(12)
C1– C1[2]	1.53(5)

[1]2/3 – *y* + *x*, 4/3 – *y*, 5/6 – *z*; [2]2 + *y* – *x*, 2 – *x*, + *z*

Table S3. Bond Angles for $AZRPbBr_3$.

	Angle (˚)
Br1^{1}–Pb^{1}–Br1^{2}	84.43(15)
Br1^{5}–Pb1–Br1^{6}	81.84(14)
Br1^{1}–Pb1–Br1^{4}	92.22(2)
Br1^{7}–Pb1–Br1^{4}	98.16(14)
Br1^{8}–Pb1–Br1^{7}	88.75(6)
Br1^{8}–Pb1–Br1^{4}	170.1(2)
Br1^{9}–Pb1–Br1^{6}	92.22(2)
Br1^{1}–Pb1–Br1^{11}	87.78(2)
Br1^{1}–Pb1–Br1^{10}	180.0
Br1^{3}–Pb1–Br1^{9}	95.57(15)
Br1^{2}–Pb1–Br1^{4}	91.25(6)
Pb1^{12}–Br1–Pb1	165.75(16)
C1^{13}–C1–C1^{14}	60.000(16)

^{1}5/3 – *y*, 4/3 – *x*, – 1/6 + *z*; ^{2}2/3 + *y* – *x*, – 2/3 + *y*, – 1/6 + *z*; ^{3}– 1/3 + *x*, 1/3 + *x* –*y*, –1/6 + *z*; ^{4}1/3 – *y* + *x*, –1/3 + *x*, 2/3 – *z*; ^{5}4/3 – *x*, 2/3 –*y*, 2/3 – *z*; ^{6}1 – *y*, + *x* – *y*, + *z*; ^{7}1/3 + *y*, 2/3 – *x* + *y*, 2/3 – *z*; ^{8}5/3 – *x*, 1/3 – *x* + *y*, 5/6 – *z*; ^{9}2/3 – *y* + *x*, 4/3 – *y*, 5/6 – *z*; ^{10} – 1/3 + *y*, – 2/3 + *x*, 5/6 – *z*; ^{11}1 + *y* – *x*, 1 – *x*, + *z*; ^{12}2/3 + *y*, 1/3 + *x*, 5/6 – *z*; ^{13}2 + *y* – *x*, 2 – *x*, + *z*; ^{14}2 – *y,* + *x* – *y*, + *z.*

DFT studies

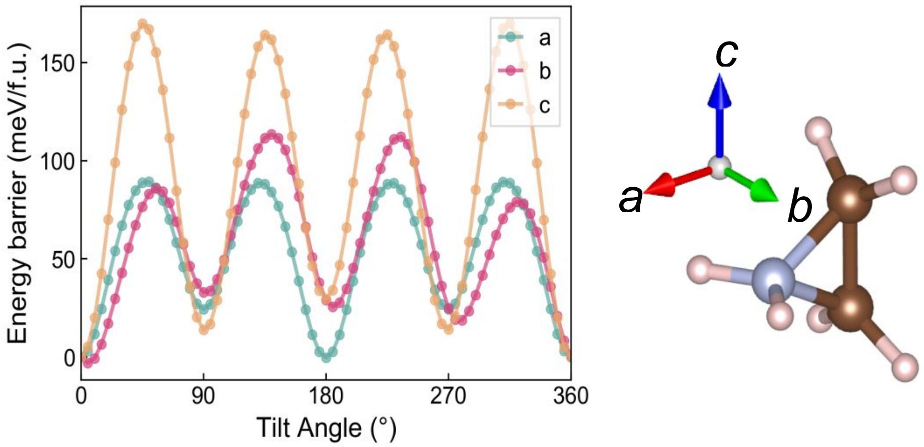

Figure S9. Relative energy of $AZRPbBr_3$ per formula unit with respect to the rotation angle of AZR^+ cation about the *a*, *b* and *c*-axes.

Raman studies

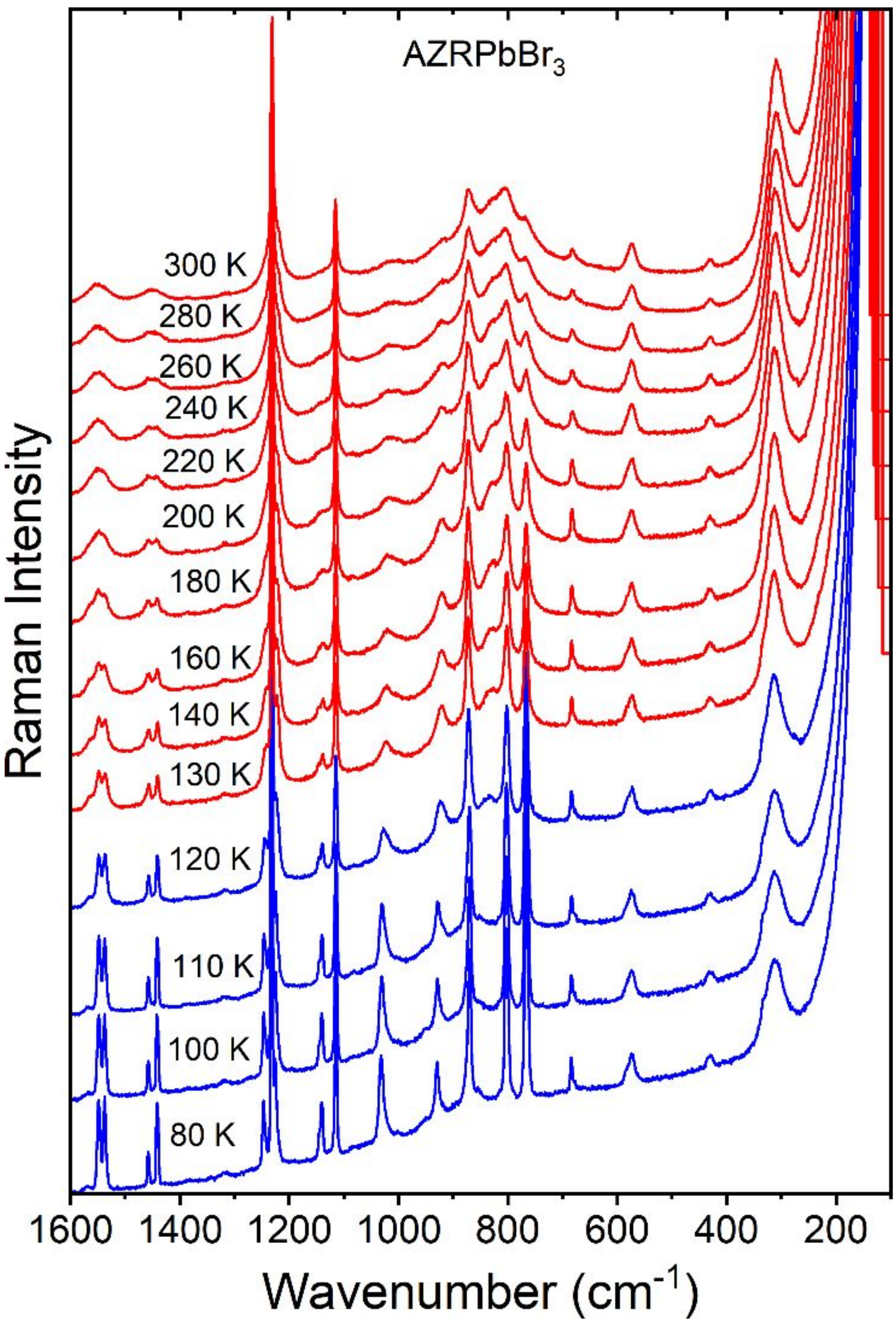

Figure S10. Temperature-dependent Raman spectra of $AZRPbBr_3$ in the 1600-100 cm^{-1} range. Bands in the 700-400 cm^{-1} range are due to the $BrCH_2CH_2PbBr_3$ impurity phase.

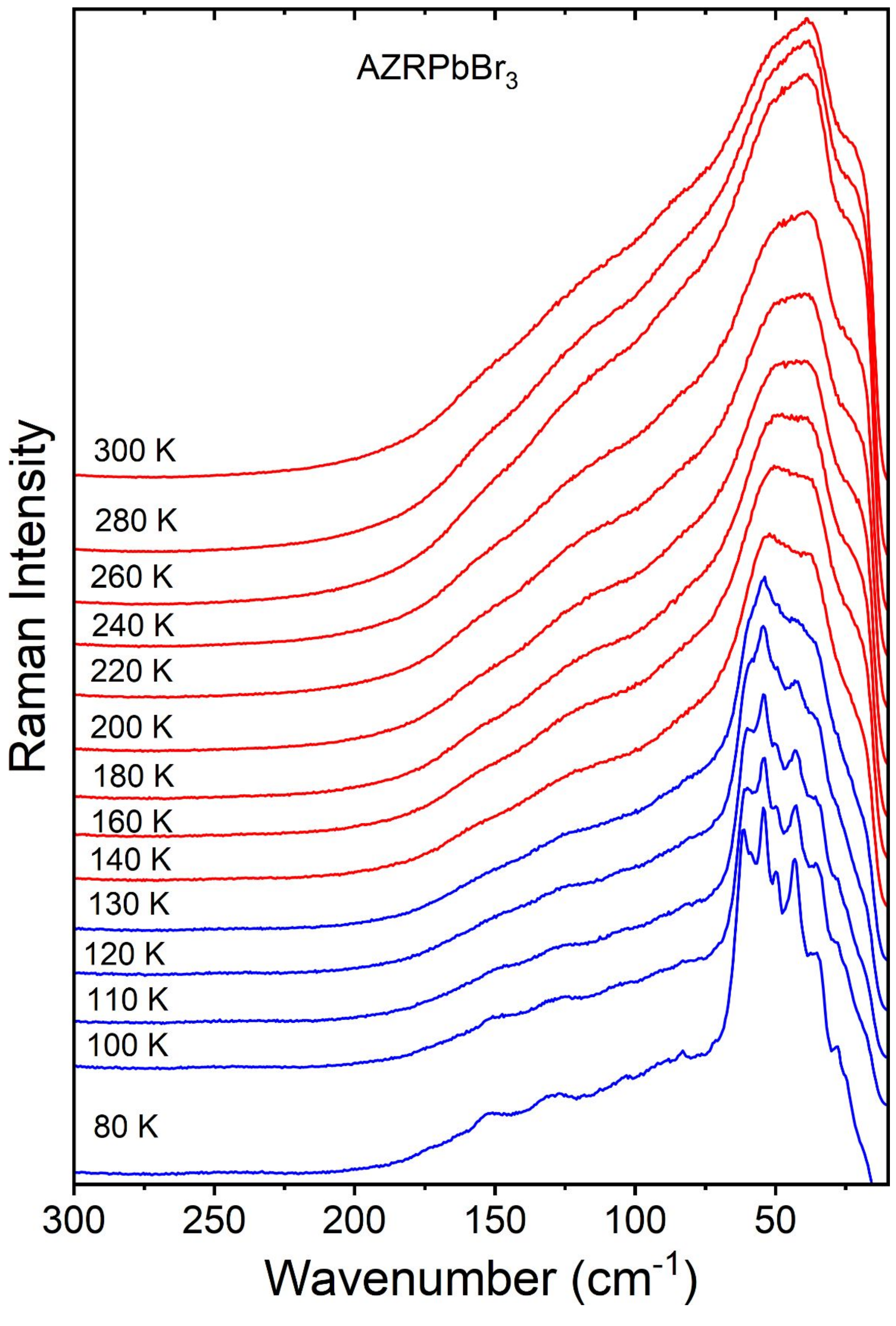

Figure S11. Temperature-dependent Raman spectra of $AZRPbBr_3$ in the 300-10 cm^{-1} range.

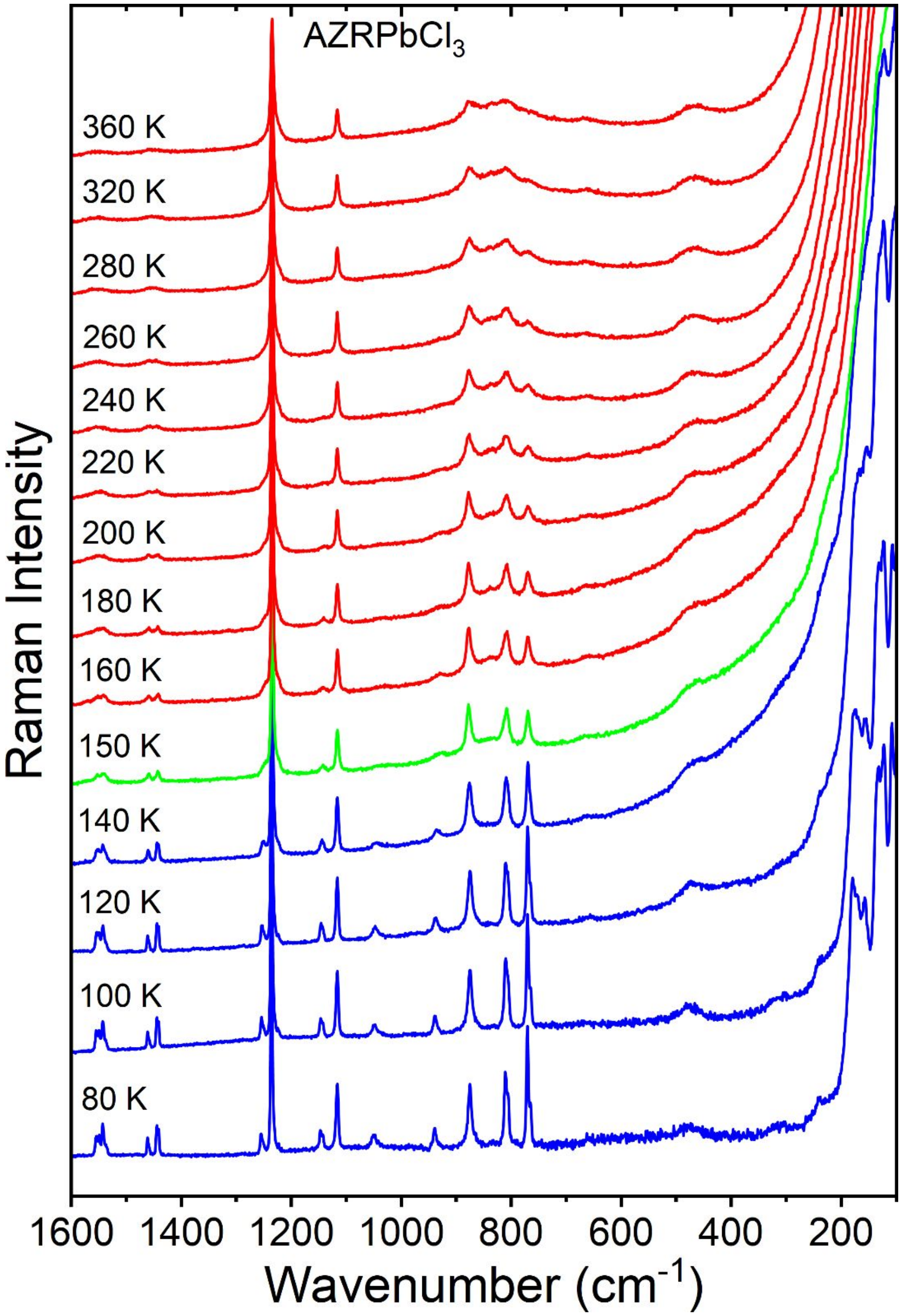

Figure S12. Temperature-dependent Raman spectra of $AZRPbCl_3$ in the 1600-100 cm^{-1} range.

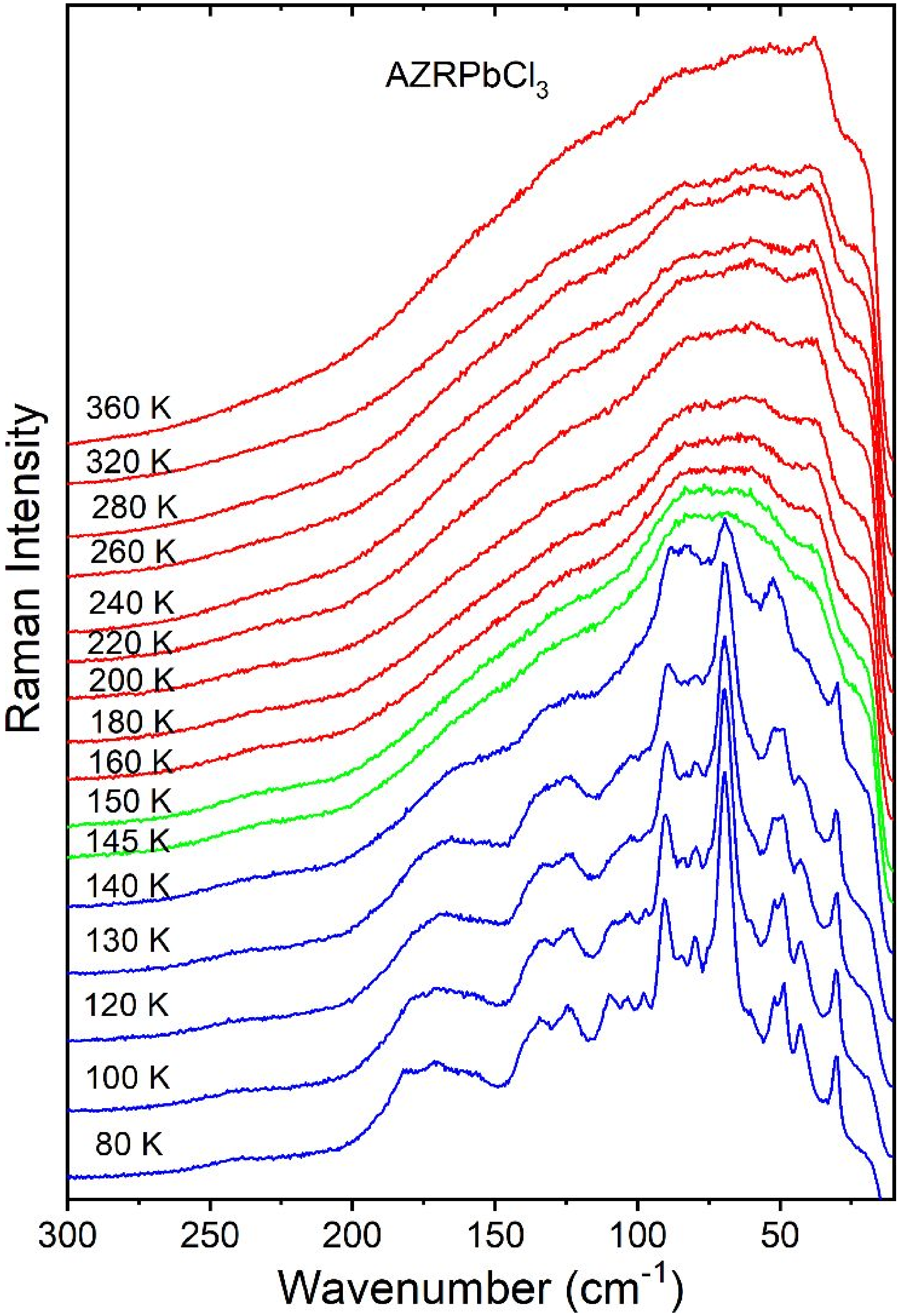

Figure S13. Temperature-dependent Raman spectra of $AZRPbCl_3$ in the 300-10 cm^{-1} range.

Table S4. Raman wavenumbers (in cm^{-1}) of $AZRPbBr_3$ and $AZRPbCl_3$ at 300 and 80 K together with the proposed assignment.[a]

$AZPbCl_3$		$AZPbBr_3$		assignment
300 K	80 K	300 K	80 K	
1554w	1550w+1543w	1550w	1548m	$\delta(NH_2)$
	1543w+1537vw		1538m	$\tau(NH_2)$
1457w	1462w+1458sh	1456w	1458w	$\delta(CH_2)$
1447sh	1445w+1441w	1444sh	1441m	$\delta(CH_2)$
	1254w	1242vw,b	1247w	$\tau(CH_2)$
1235s	1236s	1231s	1232s	ring stretch
		1220sh	1226w	$\tau(CH_2)$
	1147w+1142w	1124vw,b	1145sh+1140w	$\omega(CH_2)$
1117m	1117m	1115m	1115s	$\omega(CH_2)$
	1048w	1005w,b	1031m	$\omega(NH_2)$
	939w	920w,b	929m	$\rho(NH_2)$
875m	875m+878sh	872m	870s	ring deformation
835sh		819w		$\rho(CH_2)$
809m	810s+805m	802m	803s	ring deformation
768vw	771s+765w	765vw	767s	$\rho(CH_2)$
468m	471w	310m	314m	AZR-cage mode
	315w			T'(AZR^+)
229w	240w		249w	T'(AZR^+)+ L(AZR^+)
155sh	180m+172m+158m	153sh	174sh+152m	T'(AZR^+)+ L(AZR^+)+Pb-X stretch
121s	140sh+134m+125m	120s	130m	T'(AZR^+)+ L(AZR^+)+Pb-X stretch
	110m+104m+98m			T'(AZR^+)+ L(AZR^+)+Pb-Cl stretch
82vs	91vs+84m+80s	87sh	89m+105m	Pb-X stretch

58vs	69vs+61m	51vs	62vs+60s+54vs	Octahedra distortion (Pb-X bend)
36vs	52s+49s+43s+30s	36vs	50s+43vs+36s+28w	Octahedra twist (L(PbX_6)

[a] key: vs, very strong; s, strong; m, medium; w, weak; vw, very weak; sh, shoulder; ν, stretching; δ, bending (scissoring); ρ, rocking; ω, wagging; τ, twist; L, librational mode; T', translational mode.

Dielectric properties

We analyzed the frequency domain dielectric data of $AZRPbX_3$ compounds using the Cole-Cole equation:

$$\varepsilon^*(\omega) = \varepsilon(\infty) + \frac{\Delta\varepsilon}{1 + (\mathrm{i}\omega\tau)^{1-\alpha}} \quad \text{(S1)}$$

Here, $\varepsilon(\infty)$ is the dielectric permittivity in the high-frequency limit, $\Delta\varepsilon$ denotes the dielectric strength, τ is the mean relaxation time, and $\omega = 2\pi\nu$ is the angular measurement frequency. The relaxation width is described by the parameter $0 \leq \alpha < 1$. For $\alpha = 0$, the Cole-Cole process reduces to the Debye relaxation, which describes non-interacting electric dipoles.

Figures S14, S16 and S18 show the best fits to the experimental ε'' data using the Cole-Cole model, which allowed us to obtain the temperature dependences of the fit parameters. The temperature dependences of the determined values of τ are presented in Figures S15, S17 and S19 showing the Arrhenius behavior described by $\tau = \tau_0\exp(E_a/kT)$, where E_a and τ_0 denote the activation energy and attempt time, respectively, and k is the Boltzmann constant.

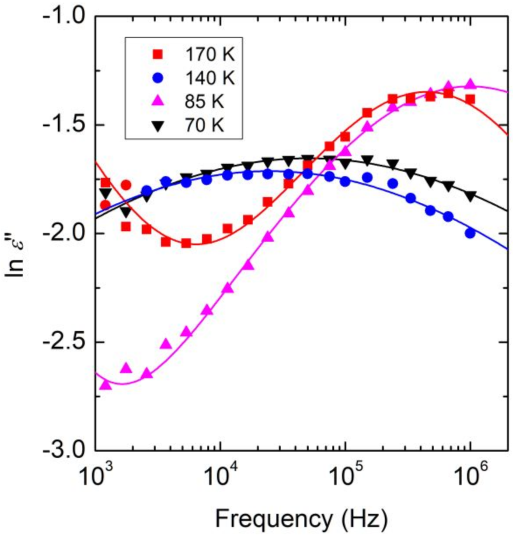

Figure S14. Frequency dependence of ε'' of AZRPbBr$_3$ for selected temperatures. Solid curves are the best fits to the Cole-Cole equation. The parameter α gradually increased from 0.4 at 170 K to 0.7 at 70 K indicating broadening of the relaxation with decreasing temperature.

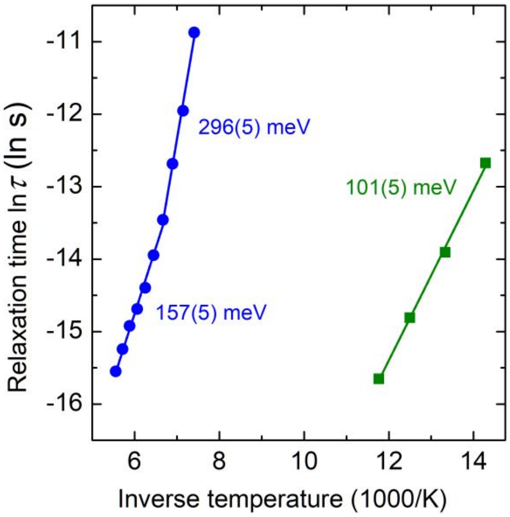

Figure S15. Arrhenius plot of the mean relaxation time of the dipolar processes of AZRPbBr$_3$. Lines are the best fits to the Arrhenius equation. Note the effect of the phase transition on the dynamics of the higher temperature dielectric relaxation.

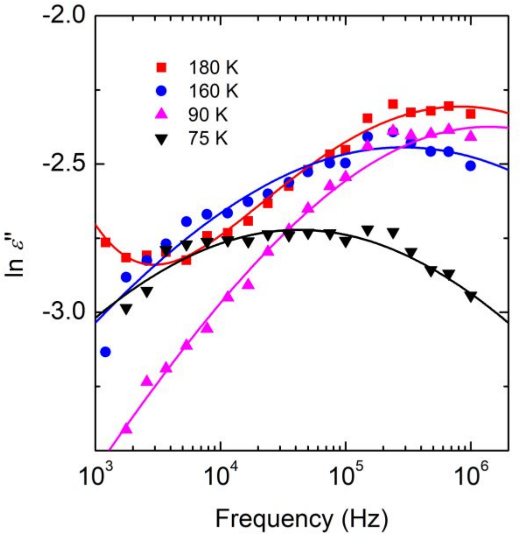

Figure S16. Frequency dependence of ε'' of $AZRPbCl_3$ for selected temperatures. Solid curves are the best fits to the Cole-Cole equation. The parameter α gradually increased from 0.5 at 180 K to 0.7 at 75 K.

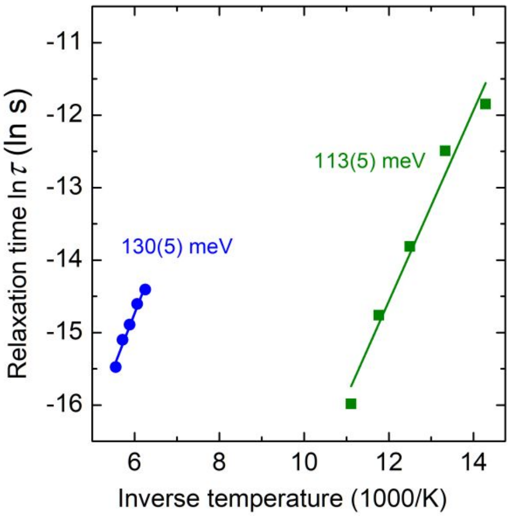

Figure S17. Arrhenius plot of the mean relaxation time of the dipolar processes of $AZRPbCl_3$. Lines are the best fits to the Arrhenius equation.

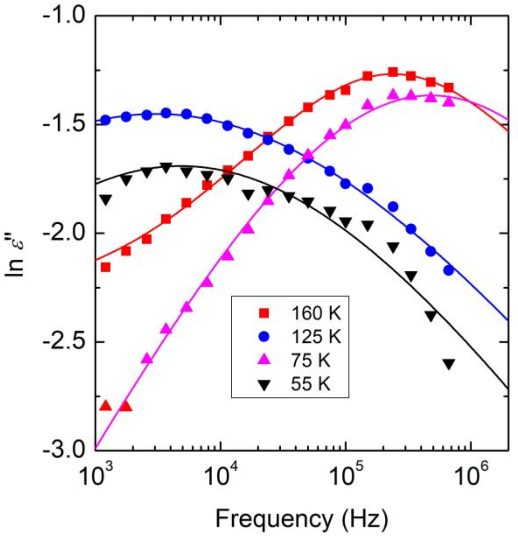

Figure S18. Frequency dependence of ε'' of AZRPbI$_3$ for selected temperatures. Solid curves are the best fits to the Cole-Cole equation. The parameter α gradually increased from 0.5 at 160 K to 0.7 at 55 K.

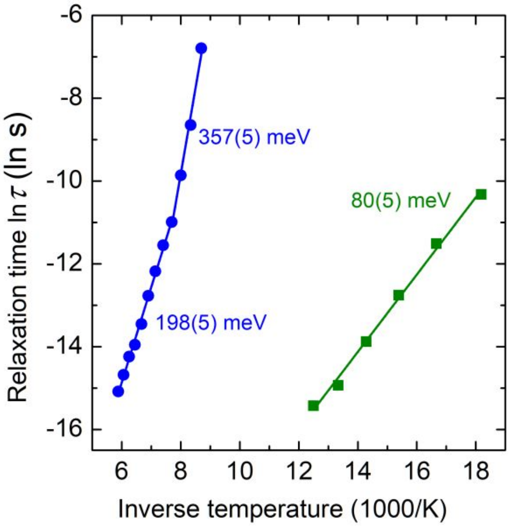

Figure S19. Arrhenius plot of the mean relaxation time of the dipolar processes of $AZRPbI_3$. Lines are the best fits to the Arrhenius equation. Note the effect of the phase transition on the dynamics of the higher temperature dielectric relaxation.

Nonlinear optical studies

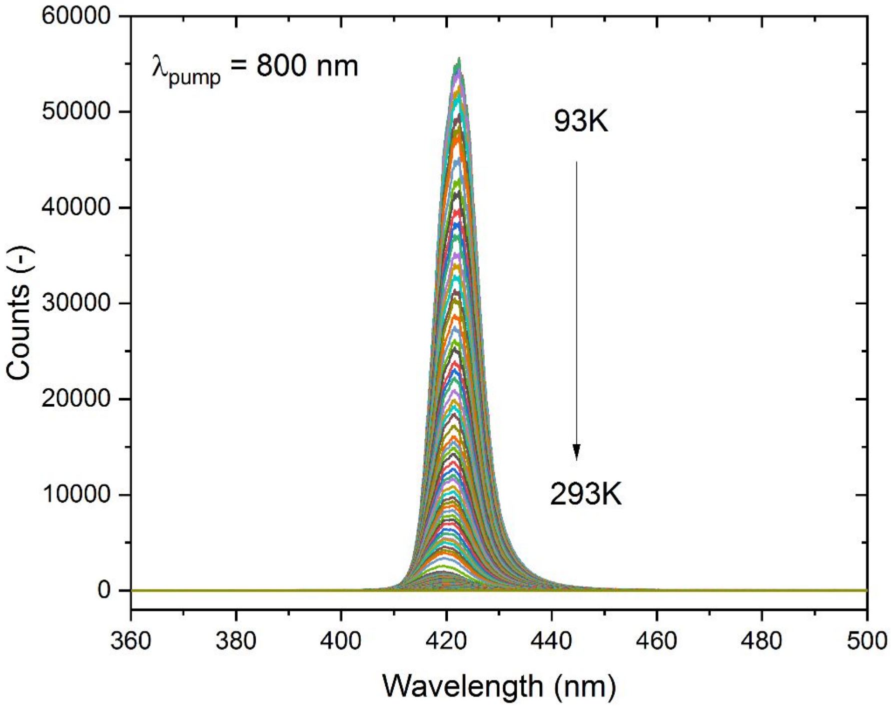

Figure S20. Overlay of experimental spectra obtained upon irradiation of $AZRPbCl_3$ with 800 nm femtosecond laser pulses during heating run from 93 K to 293 K.

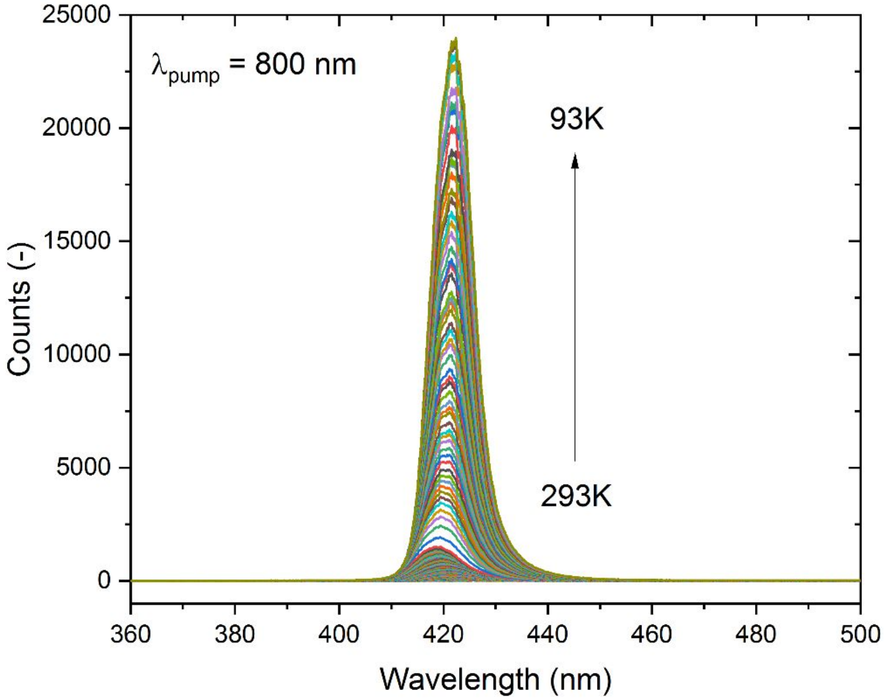

Figure S21. Overlay of experimental spectra obtained upon irradiation of $AZRPbCl_3$ with 800 nm femtosecond laser pulses during cooling run from 293 K to 93 K.

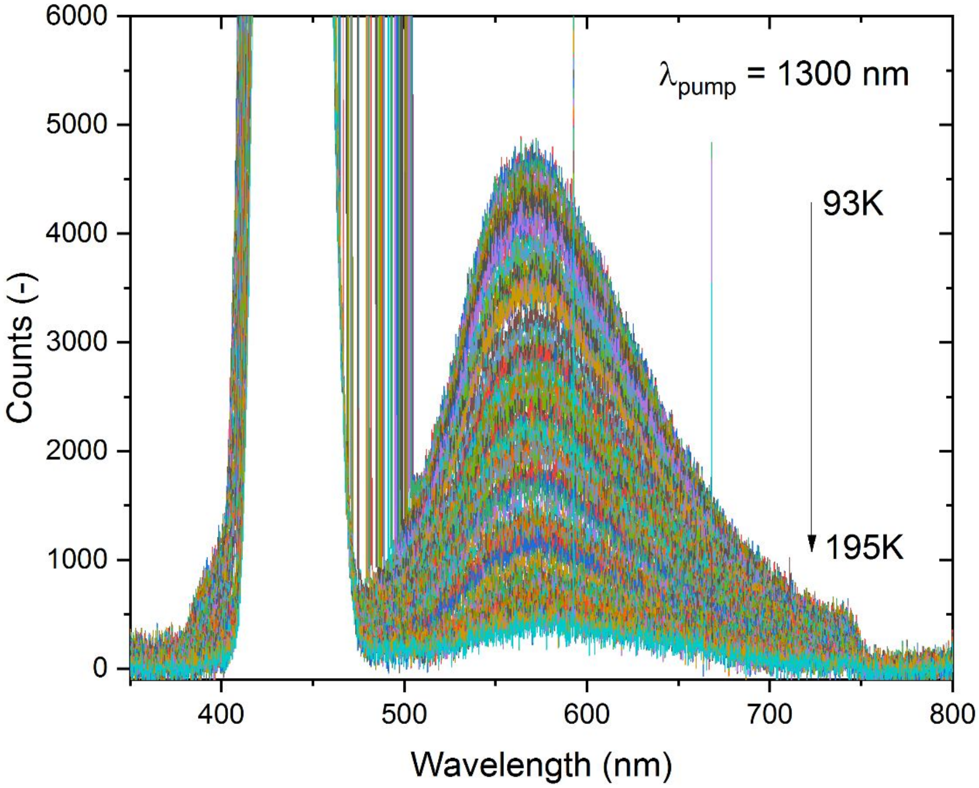

Figure S22. Overlay of experimental spectra obtained upon irradiation of $AZRPbCl_3$ with 1300 nm femtosecond laser pulses during heating run from 93 K to 195 K.

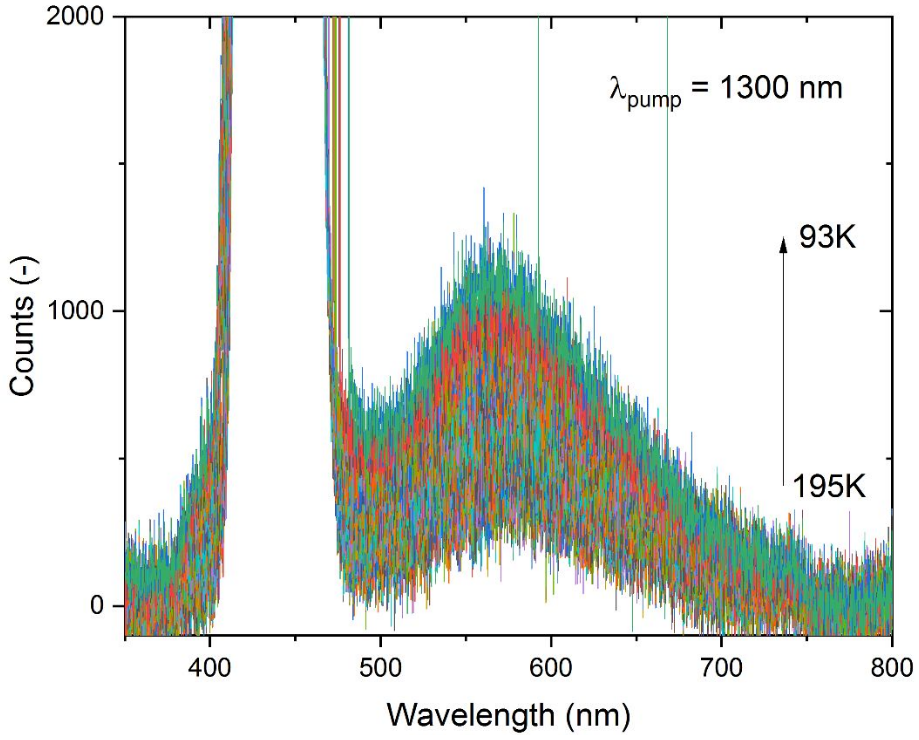

Figure S23. Overlay of experimental spectra obtained upon irradiation of AZRPbCl_3 with 1300 nm femtosecond laser pulses during cooling run from 195 K to 93 K.

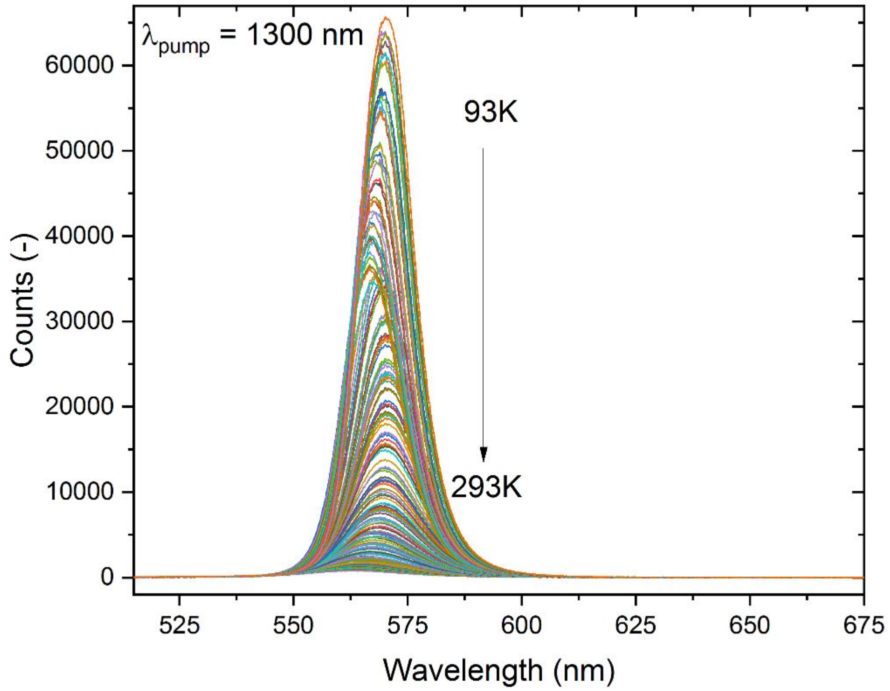

Figure S24. Overlay of experimental spectra obtained upon irradiation of AZRPbBr_3 with 1300 nm femtosecond laser pulses during heating run from 93 K to 293 K.

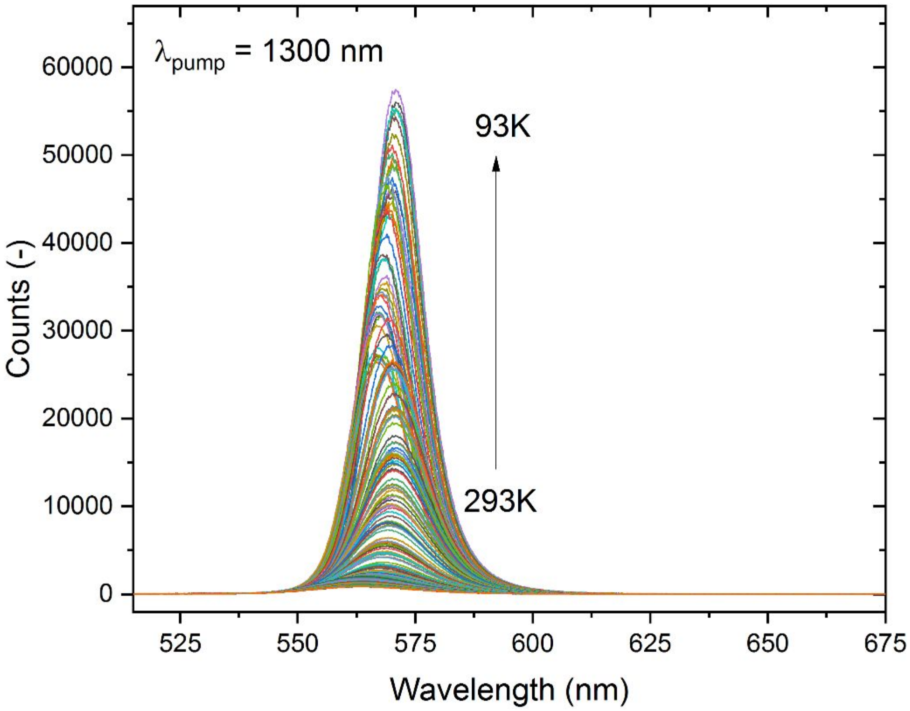

Figure S25. Overlay of experimental spectra obtained upon irradiation of $AZRPbBr_3$ with 1300 nm femtosecond laser pulses during cooling run from 293 K to 93 K.

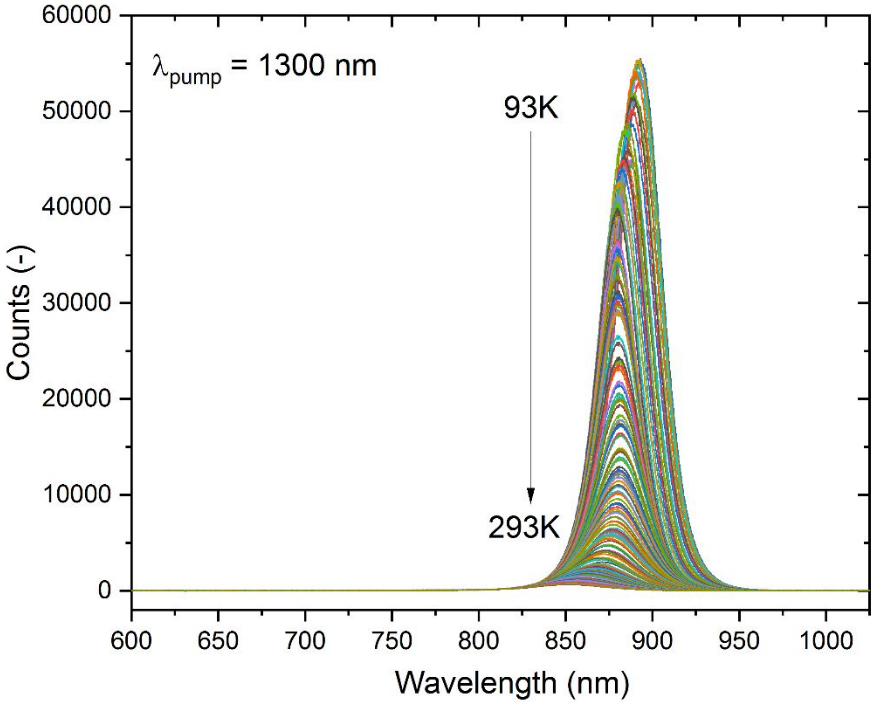

Figure S26. Overlay of experimental spectra obtained upon irradiation of $AZRPbI_3$ with 1300 nm femtosecond laser pulses during heating run from 93 K to 293 K.

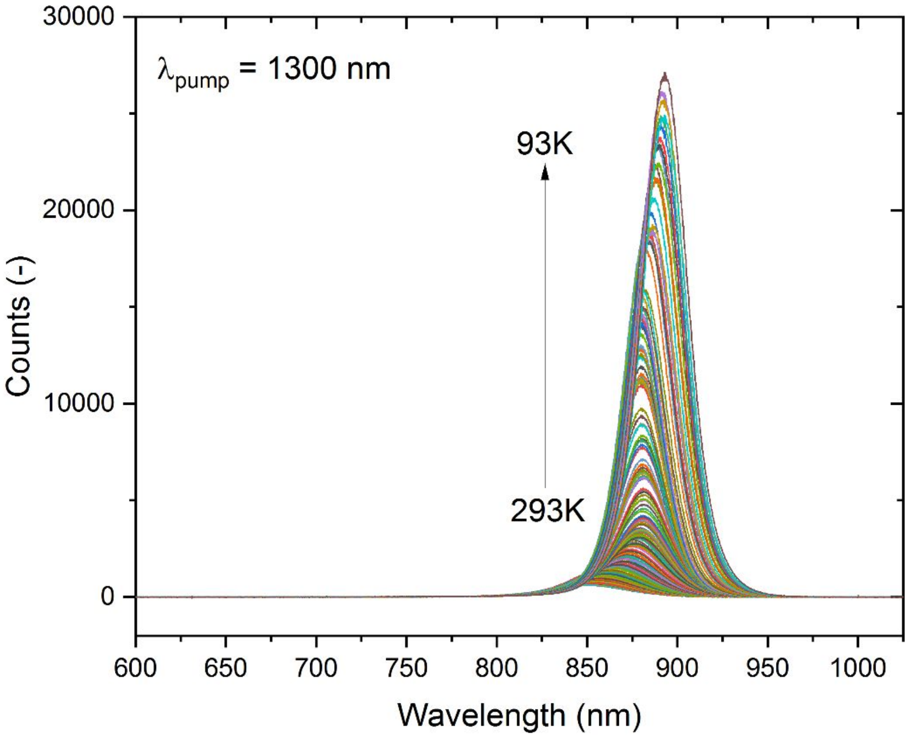

Figure S27. Overlay of experimental spectra obtained upon irradiation of AZRPbI_3 with 1300 nm femtosecond laser pulses during cooling run from 293 K to 93 K.

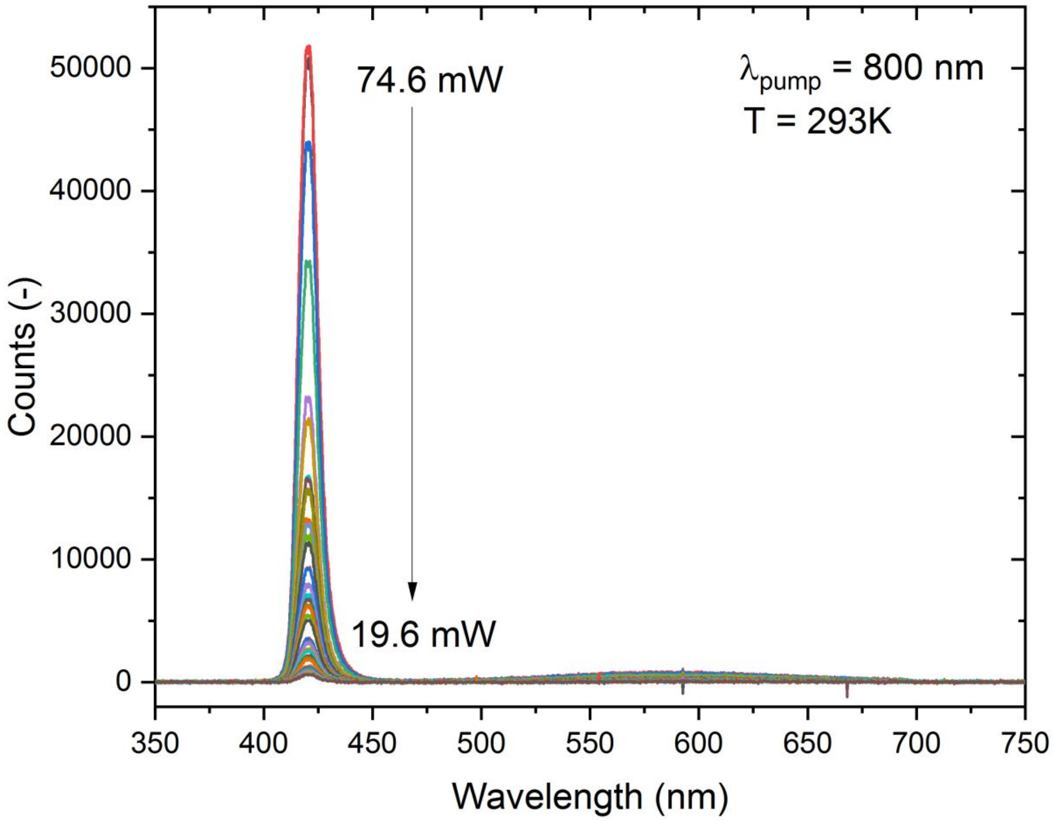

Figure S28. Overlay of experimental spectra obtained during power-dependent irradiation of $AZRPbCl_3$ with 800 nm femtosecond laser pulses.

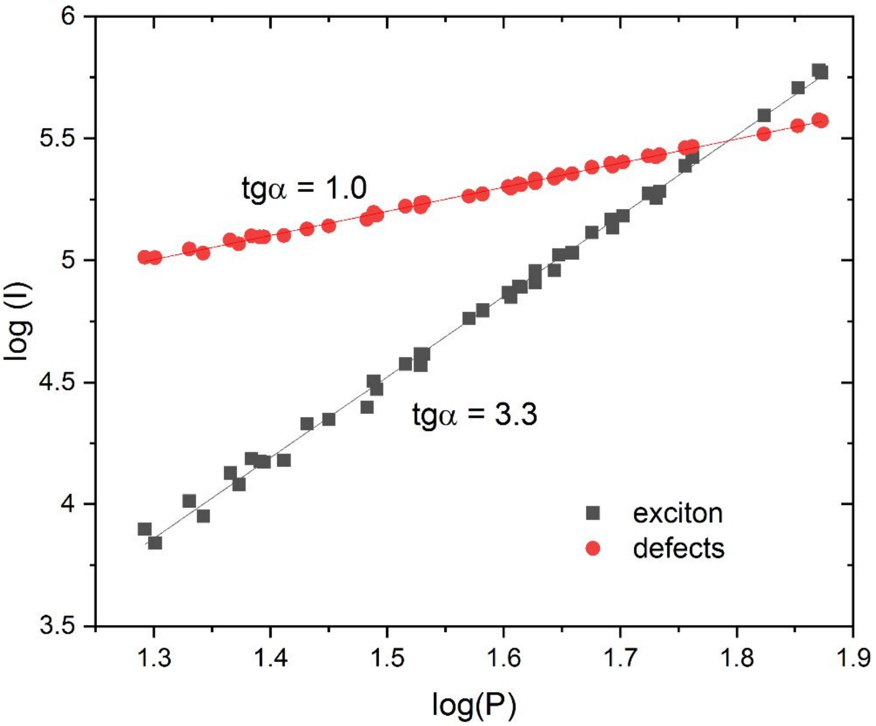

Figure S29. Log−log plot of integral intensities plotted as the function of applied laser power (800 nm femtosecond laser pulses) for $AZRPbCl_3$.

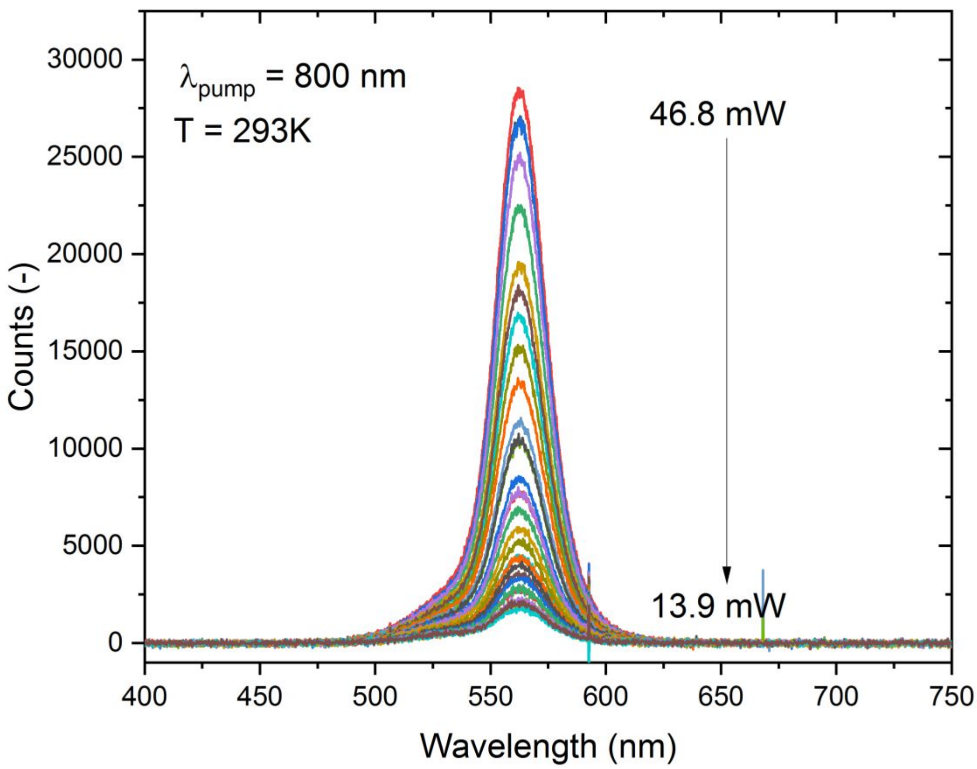

Figure S30. Overlay of experimental spectra obtained during power-dependent irradiation of $AZRPbBr_3$ with 800 nm femtosecond laser pulses.

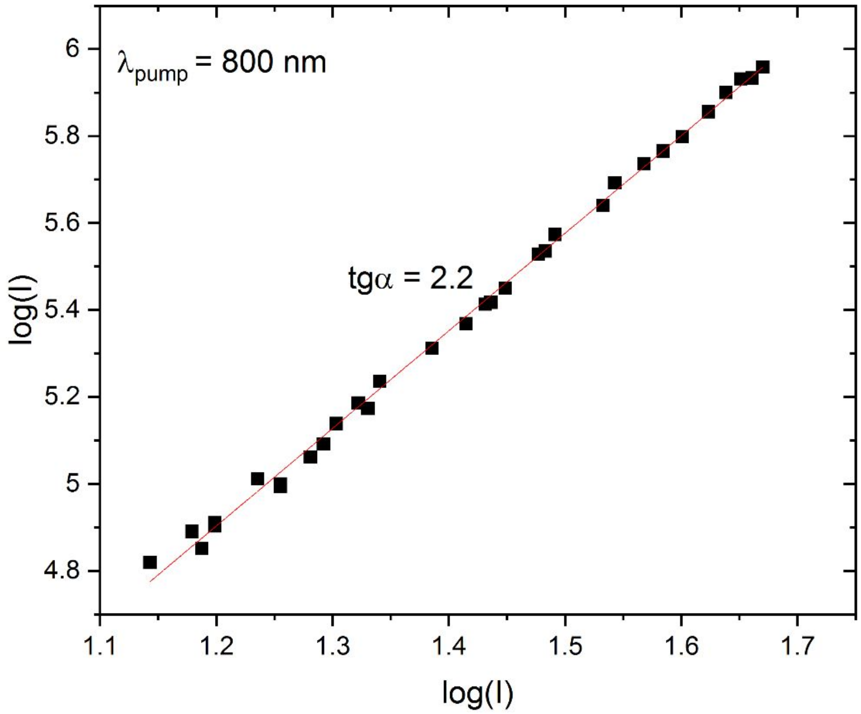

Figure S31. Log−log plot of integral intensities plotted as the function of applied laser power (800 nm femtosecond laser pulses) for $AZRPbBr_3$.

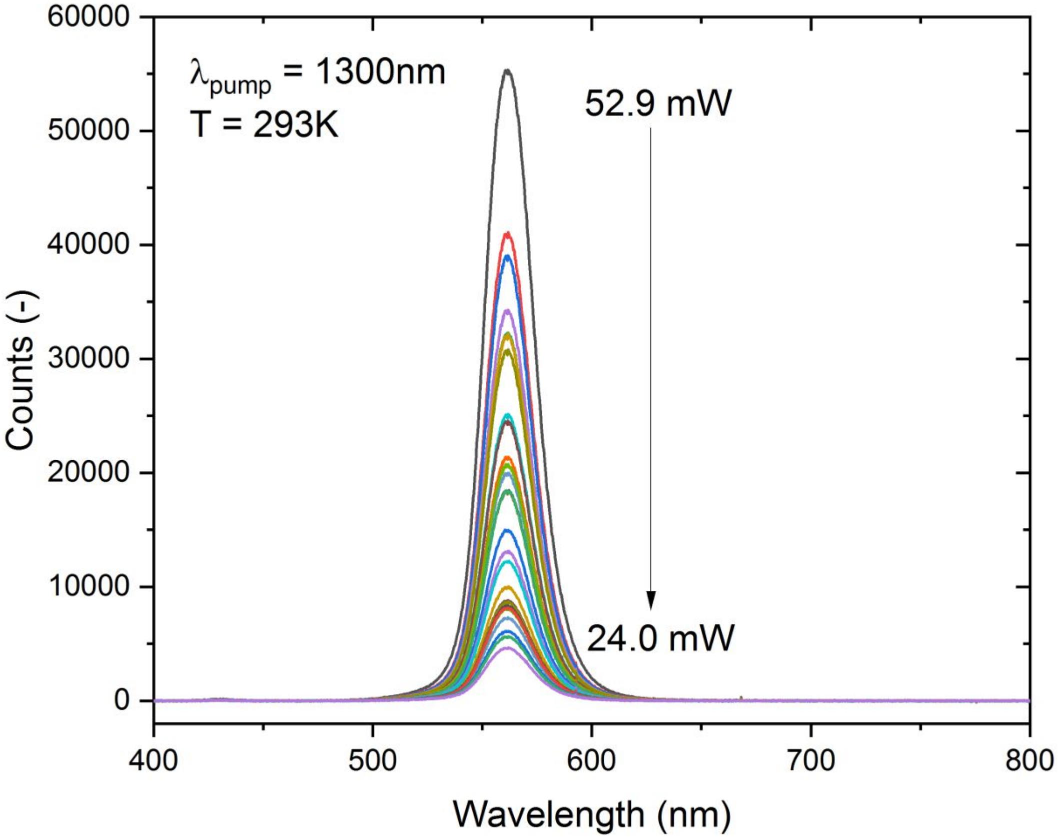

Figure S32. Overlay of experimental spectra obtained during power-dependent irradiation of $AZRPbBr_3$ with 1300 nm femtosecond laser pulses.

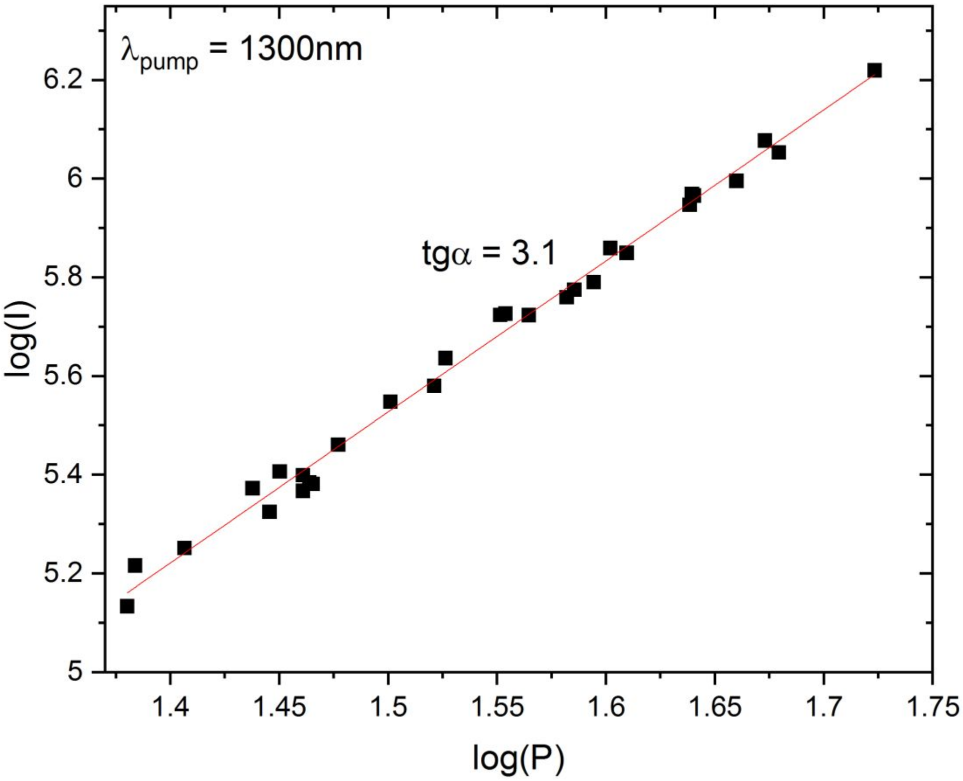

Figure S33. Log−log plot of integral intensities plotted as the function of applied laser power (1300 nm femtosecond laser pulses) for $AZRPbBr_3$.

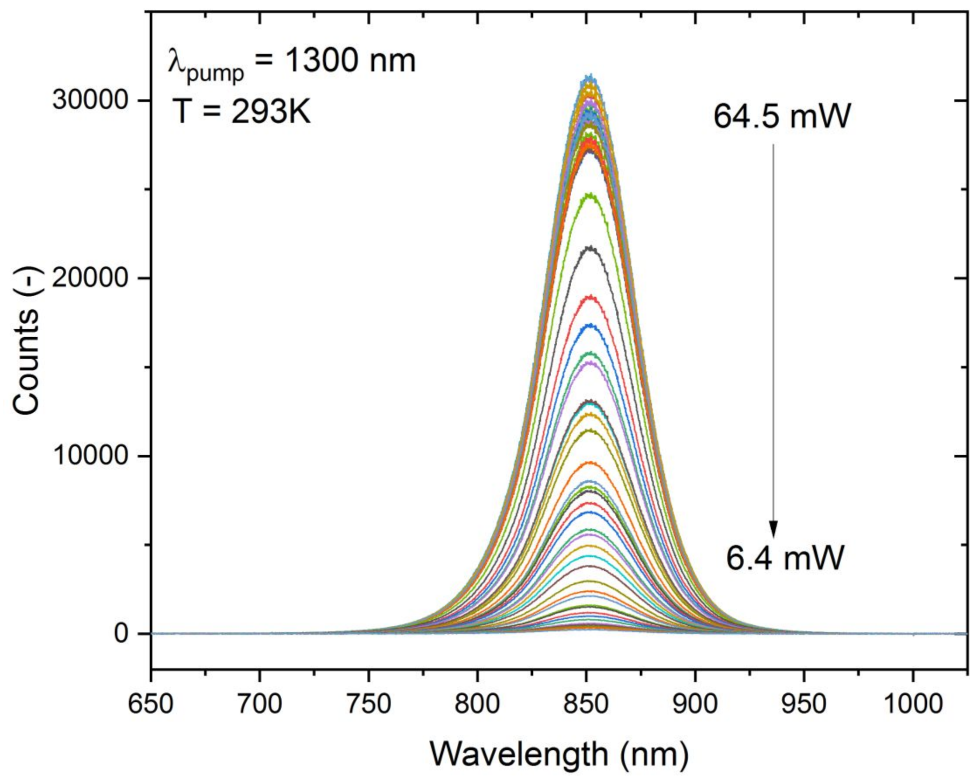

Figure S34. Overlay of experimental spectra obtained during power-dependent irradiation of $AZRPbI_3$ with 1300 nm femtosecond laser pulses.

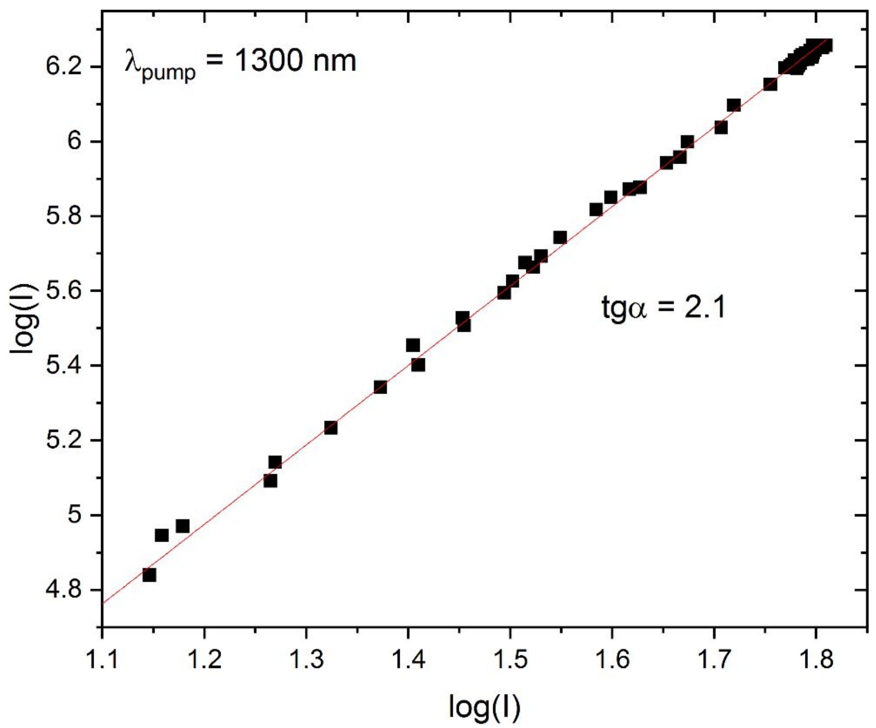

Figure S35. Log−log plot of integral intensities plotted as the function of applied laser power (1300 nm femtosecond laser pulses) for $AZRPbI_3$.

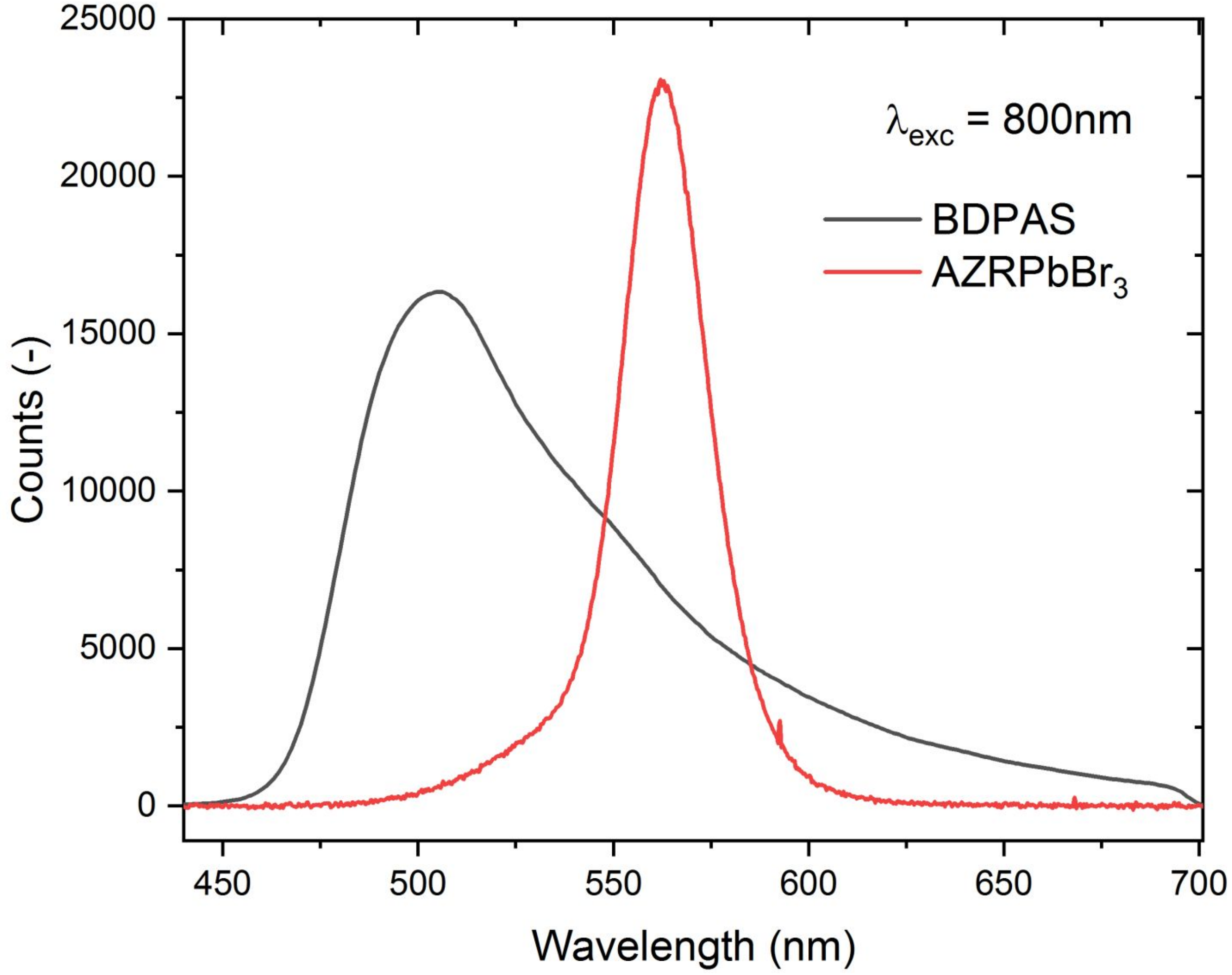

Figure S36. Emission spectra of BDPAS and $AZRPbBr_3$ obtained under 800 nm irradiation.

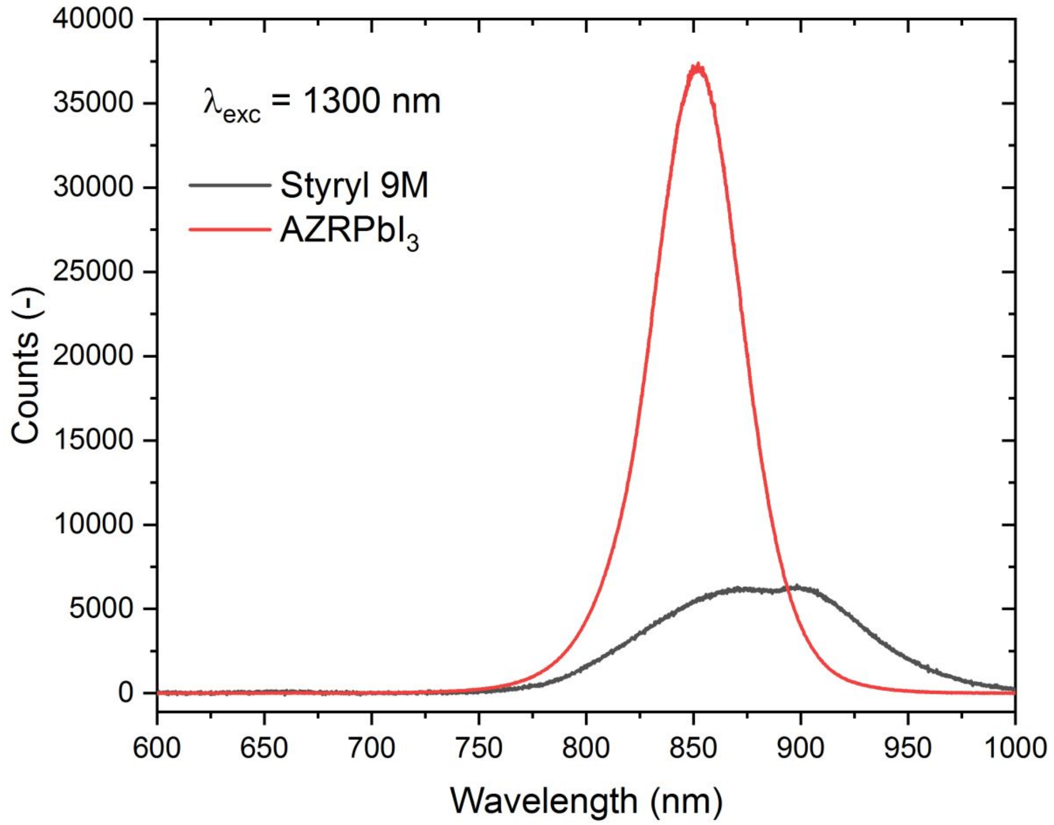

Figure S37. Emission spectra of BDPAS and AZRPbI$_3$ obtained under 1300 nm irradiation.
